# The Influence of Polysaccharides/TiO_2_ on the Model Membranes of Dipalmitoylphosphatidylglycerol and Bacterial Lipids

**DOI:** 10.3390/molecules27020343

**Published:** 2022-01-06

**Authors:** Agata Ładniak, Małgorzata Jurak, Marta Palusińska-Szysz, Agnieszka Ewa Wiącek

**Affiliations:** 1 Department of Interfacial Phenomena, Institute of Chemical Sciences, Faculty of Chemistry, Maria Curie-Skłodowska University, M. Curie-Skłodowska Sq. 3, 20-031 Lublin, Poland; malgorzata.jurak@poczta.umcs.lublin.pl (M.J.); a.wiacek@poczta.umcs.lublin.pl (A.E.W.); 2 Laboratory of X-ray Optics, Centre for Interdisciplinary Research, Faculty of Science and Health, The John Paul II Catholic University of Lublin, Konstantynów 1J, 20-708 Lublin, Poland; 3 Department of Genetics and Microbiology, Institute of Biological Sciences, Faculty of Biology and Biotechnology, Maria Curie-Skłodowska University, Akademicka 19, 20-033 Lublin, Poland; marta.palusinska-szysz@mail.umcs.pl

**Keywords:** chitosan, hyaluronic acid, titanium dioxide, DPPG, bacterial lipids, Langmuir film, π-A isotherm, Brewster angle microscopy

## Abstract

The aim of the study was to determine the bactericidal properties of popular medical, pharmaceutical, and cosmetic ingredients, namely chitosan (Ch), hyaluronic acid (HA), and titanium dioxide (TiO_2_). The characteristics presented in this paper are based on the Langmuir monolayer studies of the model biological membranes formed on subphases with these compounds or their mixtures. To prepare the Langmuir film, 1,2-dipalmitoyl-*sn*-glycero-3-phospho-rac-(1-glycerol) (DPPG) phospholipid, which is the component of most bacterial membranes, as well as biological material-lipids isolated from bacteria *Escherichia coli* and *Staphylococcus aureus* were used. The analysis of the surface pressure-mean molecular area (π-A) isotherms, compression modulus as a function of surface pressure, C_S_^−1^ = f(π), relative surface pressure as a function of time, π/π_0_ = f(t), hysteresis loops, as well as structure visualized using a Brewster angle microscope (BAM) shows clearly that Ch, HA, and TiO_2_ have antibacterial properties. Ch and TiO_2_ mostly affect *S. aureus* monolayer structure during compression. They can enhance the permeability of biological membranes leading to the bacteria cell death. In turn, HA has a greater impact on the thickness of *E. coli* film.

## 1. Introduction

The cell envelope of Gram-negative bacteria consists of two distinct layers, the outer (OM) and the inner (IM) membranes, separated by the periplasm with a thin layer of peptidoglycan. Both membrane leaflets of the IM are mostly composed of phospholipids. The OM is an asymmetric lipid bilayer containing mainly phospholipids in the inner leaflet, whereas the outer leaflet is mainly composed of lipopolysaccharide (LPS). This strict OM asymmetry is important for the proper functioning of the bacterial cell, as it constitutes a permeability barrier, preventing the penetration of toxic compounds inside of the cell [1]. Due to difficulties in studying the biological processes that take place on or within the natural membranes, resulting from the high complexity of such structures, research in this area is performed with the use of biomimetic model membranes [2]. It has been proven that their application allows the assessment of changes in the physicochemical properties of lipid layers caused by foreign substances [3].

Among the available models of biological membranes, the greatest interest has focused on biomimetic monolayer films formed using the Langmuir technique [4,5]. Although this method enables the characterization of only monomolecular films at the interface, there is a strong correlation between properties (pressure, surface area per lipid molecule, phase transition, compressibility) of mono- and bilayers prepared from cell membrane components, at surface pressures of 30–35 mN/m [6,7,8,9,10]. Owing to this, it is possible to predict, with high probability, interactions at the level of living organisms. This makes the Langmuir technique an ideal tool for obtaining information about the properties and behavior of membranes (or their components), as well as for assessing the influence of various chemical substances, e.g., biologically active, biomolecules, on the behavior of biologically important systems [8,9,10]. However, extrapolating the results obtained from such studies to actual studies of biophysical situations may require some additional considerations. To visualize the interface organization of the lipid components of a monolayer or changes in interfacial behavior resulting from the introduction of an interesting compound into the monolayer and/or subphase, and thus for a better understanding of the structure–property relation of organized films, the Langmuir technique can be easily combined with optical Brewster angle microscopy (BAM). BAM is useful for determining the structure of the tested films, detecting phase changes, emerging domains, or multi-molecular structures as well as changes in monolayer thickness and other morphological effects [11,12]. Owing to this coupling (Langmuir trough with BAM), it is possible to observe the changes occurring during compression in real-time. The analysis of these changes in relation to the π-A isotherms can provide a more precise characterization of the interactions. Knowledge about the organization of monolayers and their interactions with the components of the support and the possibility of their control at the molecular level determine the potential usefulness of the received bioproducts and enable the development of a wide range of science and technology.

Chitosan (Ch) is a linear copolymer of β-(1→4)-D-glucosamine and β-(1→4)-*N*-acetyl-D-glucosamine and has a polycationic nature in acidic solutions. The positively charged backbone of chitosan (controlled by the pH) plays a crucial role in its interaction with other macromolecules or with negatively charged or neutral phospholipids of biomembranes. Clearly, the protonated amino groups NH_3_^+^ of chitosan are involved in the mechanism of adsorption (forced by electrostatic interactions) of this positively charged polyelectrolyte on the lipid membrane of vesicles [13,14]. Contrarily, hyaluronic acid is a polyanion. It consists of glucuronic acid and *N*-acetyl-glucosamine. HA is hydrophilic, biocompatible, biodegradable, and negatively charged in a wide pH range (pKa 3–4) [15]. Its presence in thermo-reactive hydrogels leads to the improvement of viscoelastic and mucoadhesive properties. In addition, it proves that Ch and HA also can create strong interactions with inorganic substances, e.g., titanium dioxide particles (TiO_2_). Moreover, the presence of free carboxylic, amine, and hydroxyl groups at the surface of the HA and Ch films allows to bind stably the titania nanoparticles to the polymeric substrate, without the need to functionalize the nanoparticles. Moreover, these interactions lead to different physical and chemical surface adsorption, including the simple adsorption by electrostatic attraction, hydrogen bonding, and chemical binding through the formation of ester like linkage, bridging, and chelating [16,17]. TiO_2_ nanoparticles have found application in tissue engineering. They can be a component of biocomposites. Nanocomposites with bactericidal properties would be excellent materials for biomedical applications. TiO_2_ nanoparticles are being tested for use in cancer therapies. In addition, some studies confirm that both TiO_2_ and chitosan have antimicrobial (antibacterial, antifungal) properties and are also non-toxic. One of the proposed mechanisms of the antimicrobial action of Ch and TiO_2_ is based on biological disorders of the membranes of microorganisms. Therefore, it is also important to develop a mechanism of action at the molecular level, e.g., using the Langmuir monolayer technique by studying the effect of these substances on bacterial membrane components.

Knowledge about the possibility of creating or not the chemical bonds/interactions between a biomaterial and biological membrane is very important when making decisions about the specific use of a given preparation. Such an analysis is necessary regardless of the type of product prepared, and at the same time permits to direct research in order to obtain further characteristics. The effective combination of these ingredients will allow receiving a multifunctional material for applications in the field of medicine.

Therefore, we made an effort to determine whether chitosan, TiO_2_, and hyaluronic acid and/or their mixtures cause disturbances in the structure of model biological membranes, formed of the synthetic material 1,2-dipalmitoyl-*sn*-glycero-3-phospho-rac-(1-glycerol) sodium salt (DPPG) phospholipid, which is the component of most bacterial membranes, as well as biological material (lipids isolated from bacteria *Escherichia coli*, a Gram-negative organism model and *Staphylococcus aureus*, a Gram-positive organism model). This allowed us to confirm the assumption that the mechanism of the antibacterial action of these compounds and their mixtures is based on disorders of the membrane of microorganisms. That aspect was also checked by microbiological methods, using the same species of bacteria. This provided the verification of the proposed method. We assumed that the study of material isolated from microorganisms would make it possible to predict the results of microbiological tests. On the other hand, the knowledge of the mechanism of these substances interactions with membranes/monolayer is an indispensable element in the production of carriers of active substances (e.g., drugs), as it allows controlling the amount of the pharmaceuticals incorporated into the carrier (and then released from it).

## 2. Materials and Methods

### 2.1. Samples and Subphases Preparation

Chitosan (Ch; 100,000–300,000 g/mol; DD = 82 ± 2% [18] ACRōS Organics, Göteborg, Sweden) was dissolved (using homogenizer (SilentCrusher M, Heidolph, Schwabach, Germany)) in acetic acid (0.1% solution of glacial AA; 99.5–99.9%; Avantor Performance Materials, Gliwice, Poland) at a concentration of 0.1 mg/mL. Titanium dioxide (TiO_2_ particle size 10–30 nm [18]; Sigma Aldrich, Poznań, Poland) was mixed with AA in amount of 1.2 mg/mL after purification, as described previously [18,19]. Hyaluronic acid (HA; high-molecular; 1.60–1.80 MDa; 1% solution (Poznań, Poland)) was stirred with AA in proportion 0.5 mL/L. The solutions/dispersions of various combinations of these ingredients were prepared, namely: AA, AA/Ch, AA/HA, AA/TiO_2_, AA/Ch/TiO_2_, AA/HA/TiO_2_, AA/Ch/HA, AA/Ch/HA/TiO_2_, and their concentrations were simultaneously maintained. In addition, the TiO_2_-containing suspensions were placed in ultrasonic cleaner for 15 min. All samples were prepared before measurements in room temperature. As a reference, subphase deionized water from the Milli-Q system (Millipore, Burlington, MA, USA, conductivity 18.2 MΩ cm) was used.

### 2.2. Isolation of Lipids from Bacteria

Lipids from the bacterial mass of *E. coli* and *S. aureus* were isolated using the Bligh and Dyer method [20] with a chloroform-methanol-water mixture (1:2:1.8 *v*/*v*/*v*).

### 2.3. Model Bacterial Membrane Preparation (Langmuir Films)

For the preparation of model membranes, 1,2-dipalmitoyl-*sn*-glycero-3-phospho-rac-(1-glycerol) sodium salt (phospholipid DPPG) (≥99%, Sigma Aldrich, Poznań, Poland), and bacterial lipid of *Escherichia coli* and *Staphylococcus aureus* were used. DPPG was dissolved in chloroform (98.5%):methanol (99.8%) solution, in 4:1 ratio. *E. coli* lipids were dissolved in chloroform:methanol mixture (9:1) while *S. aureus* lipids in pure chloroform. All samples were prepared in concentration of 1 mg/mL. All solvents were purchased from Avantor Performance Materials (Gliwice, Poland).

### 2.4. π-A Isotherms and Stability of the Lipid Monolayers Measurements

The π-A isotherms were recorded using a double-barrier Langmuir trough (KSV NIMA, total area 783 cm^2^) placed on an anti-vibration table in a dust-reduced environment. The surface pressure was measured by the Wilhelmy plate method. Temperature of the aqueous subphase was kept constant at 20 ± 0.1 °C by a circulating water system from the thermostat (Lauda). The lipid solution was applied dropwise on subphase surface. After 10 min (necessary for solvent evaporation), compression of the lipid monolayer was followed at the constant pre-set speed of 10 mm/min (29 cm^2^/min). As a result of monolayer compression, the π-A isotherms were registered. Based on these plots, the following parameters were taking into account during analysis: A_0_, the lift-off area, defines the area per molecule at which the transition from the gas phase (G) to the expanded liquid (LE) occurs, A_lim_, the limit area, is a parameter that defines the area per molecule in a closely packed monolayer (determined by extrapolation of the linear part of the isotherm to zero surface pressure), π_coll_ is a surface pressure at the monolayer collapse. Based on data received from the π-A isotherms, the compression modulus was calculated using the formula:

$$C_{S}^{-1}=-A{\left(\frac{d\pi }{\mathrm{dA}}\right)}_{n,\;T}$$

This parameter is useful to identify the physical state of a monolayer [21,22,23] to be classified as liquid-expanded: Cs^−1^ in the range of 12.5−50 mN/m, liquid: 50−100 mN/m, liquid-condensed 100−250 mN/m, or as condensed >250 mN/m. The minima in the plots of C_S_^−1^ vs. π, on the other hand, correspond to phase transitions.

Additionally, the one-hour stability of the compressed to 35 mN/m (for DPPG) or 30 mN/m (for both bacterial extract) monolayers and compression-decompression measurements (to π = 30 or 35 mN/m; with relaxation times after compression = 1000 s) were recorded. The monolayer stability was expressed as a π/π_0_ ratio, where π-surface pressure value at given time, π_0_-surface pressure value to which the monolayer was compressed, i.e., equal 30 mN/m for *E. coli* and *S. aureus* monolayers, equal 35 mN/m for DPPG film.

### 2.5. Measurements of the Monolayer’s Morphology

The morphology of monolayers was visualized with a Brewster angle microscope (BAM; Accurion, Goettingen, Germany) equipped with a 50 mW laser emitting *p*-polarized light at a wavelength of 658 nm, a polarizer, an analyzer, and a CCD camera. The BAM apparatus was coupled with the Langmuir trough. The procedure of monolayer formation was identical to that described above. BAM images presented in this paper show monolayer fragments of 360 × 200 μm^2^. Due to the application of appropriate parameters of the polarizer and analyzer, the aqueous solution (without the monolayer) at Brewster’s angle takes a shade of black, and the lipid domains appearing in this field take a shade of gray, which makes it possible to determine the thickness of the monolayer. Moreover, owing to the single-layer optical model [24,25], it is also possible to estimate the thickness of the phospholipid layer formed during the compression.

### 2.6. Microbiological Tests

Antimicrobial activity of chitosan Ch (AA/Ch), AA/TiO_2__,_ AA/HA, AA/Ch/HA, AA/Ch/TiO_2_, AA/HA/TiO_2_, and AA/Ch/HA/TiO_2_ against *Escherichia coli* and *Staphylococcus aureus* was determined using a colony forming unit (CFU)-counting assay and Live/Dead staining combined with fluorescent intensity measurements. *E. coli* (ATCC 25922) and *S. aureus* (ATCC 25923) were grown in 5 mL Luria-Bertani (LB) medium at 37 °C with agitation at 200 r.p.m.

A bacterial suspension with OD_600_ of 0.1 was diluted 10^−4^-fold in LB medium. Next, 5 μL of the last dilution of the bacterial culture was transferred into a sterile Eppendorf tube, mixed with 5 μL of LB medium (control) or suspension of the appropriate compound (final concentration was the same as for subphase, e.g., AA: 0.1%, Ch: 0.1 mg/mL, TiO_2_: 1.2 mg/mL, HA: 0.5 mL/L (*v*/*v*)), and incubated for 1 h at 37 °C. The mixtures were transferred onto agar plates and the colonies were counted after 1 day of incubation at 37 °C (BTL). The controls defined the total (100%) survival of *E. coli* and *S. aureus* cells incubated in identical conditions but without compound addition. Results from the antimicrobial assay represent the mean of three independent experiments, with three replicates per experiment.

Hence, 100 μL of the test compounds (final concentrations AA: 0.1%, Ch: 0.1 mg/mL, TiO_2_: 1.2 mg/mL, HA: 0.5 mL/L (*v*/*v*)) were added to 100 μL of bacterial suspension (*E. coli*, *S. aureus*) with OD = 0.1. After one hour of incubation of all samples at 37 °C, the samples containing TiO_2_ were centrifuged at 500× *g*, 1 min, TR (Labnet Prism R). The supernatants were transferred into a sterile Eppendorf tube and stained using the Live/Dead BacLight bacteria viability assay kit (ThermoFisher, Waltham, Massachusetts, United States). The Live/Dead BacLight bacteria viability kit allows to discriminate viable bacterial cells from membrane compromised dead cells. The BacLight is composed of the nucleic acid-binding stains SYTO 9 and propidium iodide (PI) which differ in their spectral characteristics, as well as in their ability to penetrate bacterial cells. SYTO 9 penetrates bacterial membranes resulting in a green fluorescing signal, whereas PI is membrane impermeant and penetrates cells with damaged membranes only. Live/Dead staining of the bacteria was performed by incubation for 15 min in Live/Dead staining solution (5 μM Syto9 and 30 μM PI in 3% DMSO) in RT.

Images of the bacteria were collected using an Axiovert 200 M confocal microscope with an LSM 5 PASCAL scanning head (Carl Zeiss, Jena, Germany). The Live/Dead test was performed on the basis of 20 images of each sample. Images were captured with AxioVision 4.8 software (Carl Zeiss) in the multichannel fluorescence technique, with the AxioCam HR3 camera, using 470 nm and 546 nm filters for the green and red channels, respectively, with the same exposure time for each pair of images. Analyses were performed using ImageJ 1.50i (Wayne Rasband, National Institutes of Health, Kensington, MD, USA). The sum of the lighting values was analyzed separately for each channel in a pair of images, which corresponds to the percentage of live and dead bacteria.

## 3. Results

### 3.1. Effect of Ch, HA, TiO_2_ on the DPPG Model Membranes

#### 3.1.1. Behavior of the DPPG Monolayer during Compression

In general, the basis for the analysis of the interactions between the components within the monolayer, as well as the components constituting the film and those present in the subphase, is the dependence of changes in surface pressure (π) as a function of the area per one molecule in the monolayer (A) (called π-A isotherms), recorded during compression measurements. Surface pressure, defined as the difference between the water surface tension without and with the presence of the Langmuir film, is measured by the Wilhelmy plate method. During this process, the physical state of the monolayer changes (gaseous (G), expanded liquid (LE), condensed liquid (LC), solid (S) are distinguished), and is influenced by the intermolecular interactions occurring at the interface, whose strength and range change due to approaching the molecules to each other. Particular orientations and packing of molecules on the subphase surface correspond to the specific states. On the other hand, under high surface pressure, the monolayer may bend or collapse, which is manifested by a sudden drop/change in the surface pressure. Therefore, the analysis of the shape, course, and position of the π-A curves provides information on the phase transitions, monolayer phase state, and its organization and stability (packing density and molecular orientation or conformation).

**Figure 1:**
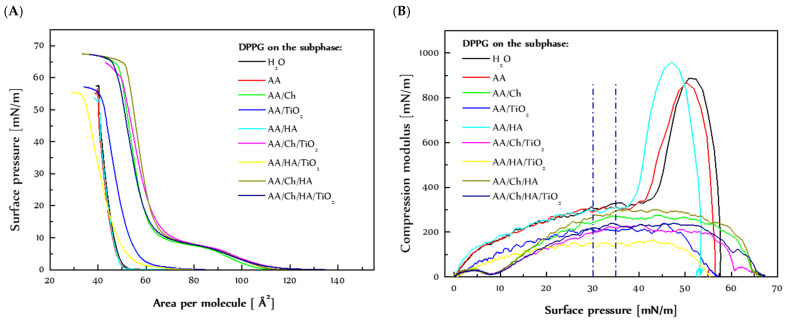
(**A**) π = f(A) isotherms obtained by means of the Langmuir technique and determined based on them (**B**) C_S_^−1^ = f(π) relationships for the DPPG monolayer on individual subphases.

The π-A isotherm formed during compression of the DPPG monolayer on the water subphase (H_2_O) was called a reference (Figure 1). Additionally, for better visualization of results, the values of A_0_ and A_lim_ parameters are presented in Table 1.

The π-A isotherms of DPPG monolayers registered on subphases containing chitosan, TiO_2_, and/or hyaluronic acid had a completely different course in relation to the reference (Figure 1A). Interestingly, the use of acetic acid (AA) and acetic acid with the addition of hyaluronic acid (AA/HA) as a subphase for the lipid films did not cause the visible changes during their compression. The course/shape of isotherms for the DPPG monolayers on AA and AA/HA was the same as for the reference isotherm. Only the curves were shifted towards slightly smaller areas per molecule (in relation to the isotherm of DPPG on H_2_O) and collapsed at slightly lower surface pressure values.

The A_0_ parameter (Table 1) increases with the addition of further components to the subphase, which indicates that the chitosan and TiO_2_ get between the DPPG molecules weakening the attraction forces between the phospholipid molecules. Only the use of acetic acid and acetic acid with the addition of hyaluronic acid has the opposite effect, that is, it slightly enhances the attraction forces between the DPPG molecules. This conclusion also arrived from analysis of limit area values (A_lim_). In addition, the limit area per molecule in the monolayer grows with the increasing amounts of components in the subphase. This means that even at high compression, subphase components (excluding AA, and AA/HA) mostly affect the monolayer behavior.

The DPPG phospholipid (pKa 3.5–4) bears the negative charge due to the presence of the negatively charged PO_4_^−^ group and non-deprotonated glycerol [22]. However, in the DPPG monolayers on AA and AA/HA, despite the presence of acetic acid and hyaluronic acid in the subphase (pH 3.4, 3.7 respectively), the arrangement of the DPPG molecules during compression did not change. The G/LE phase transition (A_0_ parameter) was not shifted significantly, and the LC/S transition was not lost (remain visible; see graph C_S_^−1^ = f(π), Figure 1)). Moreover, the AA and HA presence did not affect the A_lim_ values which means that those substances did not substantially affect the organization of the tightly packed DPPG monolayer. Under applied conditions, a strong intermolecular hydrogen bonding between the glycerol hydroxyl and the phosphate of the neighboring lipid molecules can prevail, resulting in dense packing and rigidity of the monolayer [22].

The most significant changes in the course of the π-A isotherms were noted after additional introduction of TiO_2_ to those (AA, AA/HA) subphases. In the case of TiO_2_-containing dispersions (AA/TiO_2_, AA/HA/TiO_2_), the curves moved towards larger values of area per molecule, suggesting a weakening of the attraction forces between the DPPG molecules. In addition, TiO_2_ caused a milder increase in surface pressure when the condensed DPPG domains formed during transition from the gas to expanded liquid phase and then to the condensed liquid phase. Moreover, the monolayers collapsed at a surface pressure similar to that of the monolayer spread on water but probably according to a different mechanism induced by TiO_2_.

The negatively charged heads of the phospholipid create favorable conditions for interaction with positively charged TiO_2_ particles. TiO_2_ penetrates between DPPG molecules at the beginning of compression, causing a reduction in the available area at the interface, thus accelerating the G-LE phase transition. However, the slight difference of π_coll_ values to the reference suggests that the TiO_2_ particles could be pushed out of the monolayer at high surface pressures. On the other hand, the change in the shape of the isotherms for DPPG monolayers on the TiO_2_-containing subphases is indicative of DPPG-TiO_2_ interactions, and thus TiO_2_ particles may be located just below the surface of the lipid film. The close presence of TiO_2_ imposes a different arrangement of both the charged hydrophilic heads and the hydrophobic chains of DPPG molecules at the interface. The DPPG molecule consists of two saturated chains which, under high surface pressure, due to the lack of spatial restrictions, position themselves vertically in relation to the liquid surface. In turn, the head of the phospholipid has both a negatively charged phosphate group and glycerol, which is a very weak acid (pKa = 14.4), occurring in the protonated form. The appearance of a positive charge (TiO_2_) in the vicinity may determine the formation of strong interaction with PO_4_^−^, and thus change the arrangement of the entire fragment of the molecule.

Moreover, the analysis of the C_s_^−1^ = f(π) relationship (Figure 1B) suggests that the addition of chitosan and TiO_2_ to the subphase caused deviations from the typical course of the curve. However, acetic acid and hyaluronic acid caused only shifts, but allowed maintaining the correct shape.

On the typical π-A isotherms for DPPG, the LE-LC phase transition region is not visible because in the DPPG monolayers the condensed domains are formed at lower values of surface pressure [26]. In addition, the compression modulus value at approximately 45 mN/m proves that the state of the DPPG monolayer changes from the condensed liquid phase to a solid. The lack of LE-LC phase transition if the acetic acid or acetic acid with the addition of hyaluronic acid subphases are used confirms that these components do not affect the DPPG monolayer properties. They only affect the value at which the LC-S phase transition occurs.

The presence of TiO_2_ causes a complete disintegration of the DPPG monolayer, which manifests itself as the disappearance of the LC-S phase transition and difficulty in the formation of a compact film. The compression modulus value has been significantly reduced, which means that in the presence of TiO_2_ the DPPG monolayers exist in the less packed and ordered state. This drastic reduction in packing suggests great weakness of the attraction forces between DPPG molecules, causing the monolayer to be highly elastic independently of the surface pressure values.

Drastic changes in the course of the π-A isotherms occurred in the case of chitosan-containing subphases. The isotherms shift towards larger areas as compared to the reference which indicates the penetration of chitosan into the DPPG monolayer, thereby causing increasing repulsive Coulombic forces between negatively charged DPPG headgroups. This gives rise to looser molecular packing in DPPG monolayers, where the LE-LC phase transition is observed [22]. After compression of the monolayers, the π-A isotherms registered for chitosan-containing subphases were collapsed at much higher values of surface pressure than that of the monolayer on water. This can suggest its presence in a closely packed monolayer.

As mentioned above, the introduction of chitosan into the subphase causes the appearance of the LE-LC phase transition region, which does not occur for the DPPG monolayer on water. This denotes that the Ch presence slows the creation of condensed domains in the DPPG monolayers. As an effect, the LE-LC phase transition is revealed. Similar results were obtained by Piosik et al. [27], who examined the effect of nanoparticles with a Fe_3_O_4_ core and a chitosan crown on the behavior and structure of the DPPG monolayer. The presence of the Fe_3_O_4_-Ch nanoparticles in the water also causes the appearance of the liquid expanded phase between the gaseous state and liquid condensed phase. Additionally, the shift of the π-A isotherms of DPPG in the presence Fe_3_O_4_-Ch and thus the expansion increases with the rising concentration of the Fe_3_O_4_-Ch nanoparticles, indicating the adsorption of the investigated nanoparticles into the phospholipid monolayers, which were probably located preferably into loosen monolayer areas in the liquid expanded phase.

Referring again to our chitosan-containing subphases, the graph shows a plateau which is indicative of LE-LC phase transition (Figure 1A). The precise value of the LE-LC phase transition pressure can be read based on the dependency of the compression modulus as a function of the surface pressure. The graph (Figure 1B) shows a minimum in the course of the function, dividing the graph into two maxima that correspond to the expanded liquid (LE) and the condensed liquid (LC). The presence of chitosan contributed to the fluidity of the DPPG monolayers, keeping them in the state of LC at high values of surface pressure. On the other hand, DPPG monolayer on the chitosan-containing subphases showed slightly more stability than the reference one. They were collapsed at much higher surface pressure values. The same observations were obtained by [26], where the presence of Fe_3_O_4_-Ch also caused a decrease of the maximal C_S_^−1^ value associated with the disappearance of the solid state at the DPPG final compression stage. In addition, it is noticeable to reduce the monolayer packing after an additional introduction of TiO_2_ into the Ch-containing subphases (lower values of the compression modulus were received). Ultimately, the shape/course both the π-A isotherms and the C_S_^−1^ = f(π) relationships was very similar to those obtained for DPPC monolayers [28]. This means that as a result of the interactions of Ch with DPPG molecules, similar conditions occurred during compression. DPPC has the same hydrophobic part as DPPG but differs in the hydrophilic part. DPPC is zwitterionic, i.e., it has two differentially charged groups, PO_4_^−^ and -N(CH_3_)_3_^+^. On the one hand, this may suggest that (I) Ch penetrates between DPPG molecules, interacting with the phosphate groups, thus reducing the charge of the hydrophilic heads, (II) Ch causes the vertical orientation of the DPPG molecules at the interface, (III) Ch remains in the monolayer even during its high compression (A_lim_ value was significantly higher than the reference), (IV) Ch determines some kind of stabilization of the lipid film (π_coll_ was much higher than the reference), but at the same time (V) Ch increases the fluidization of the film (keeping it in the LC state). This observation may be a result of separation of the phosphate-groups by the positively charged Ch in the DPPG headgroup region which prevents a formation of rigid and tightly packed monolayer. As a consequence, the LE-LC phase transition is revealed.

In order to more exactly characterize the interactions between the subphase components (TiO_2_, HA, and Ch) and DPPG molecules, the stability of DPPG monolayers (expressed as π/π_0_ = f(t)) previously compressed to π = 35 mN/m, was tested.

#### 3.1.2. Stability of the DPPG Monolayer

The lateral pressure on the monolayer during its compression forces the appropriate behaviour and orientation of DPPG molecules. When compression is stopped (and thus surface pressure does not increase), phospholipid molecules reorganize and any changes in surface pressure during relaxation indicate their different responses to the mechanical compression. Among other things, the changes taking place may also suggest the loss of lipid material from the interface. This in turn could be confirmed by the compression-decompression measurements. The observation of the stability of the monolayers over time, combined with the analysis of hysteresis loops, provides detailed information on the interactions between the components of the subphase and the model membrane.

**Figure 2:**
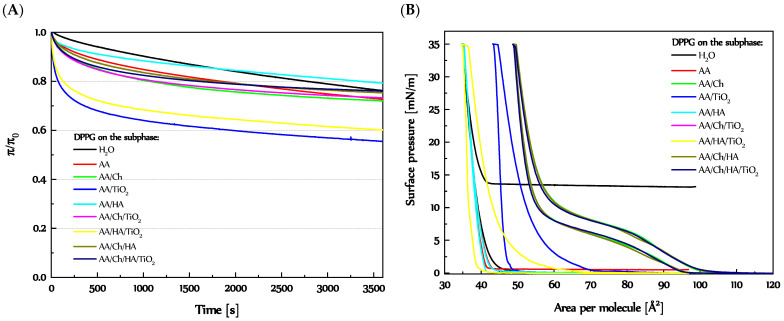
(**A**) π/π_0_ = f(t) (more details Figure S1) and (**B**) hysteresis loops obtained by compression and decompression of the DPPG monolayers on the individual subphases.

Figure 2A shows changes of a relative surface pressure over one hour relaxation of DPPG monolayers, previously compressed to 35 mN/m on different subphases. As can be seen, a rapid decrease of π/π_0_ ratio took place within 15 min for all cases. After that time, the DPPG monolayer showed comparatively constant values of relative surface pressure. The trend of π/π_0_ ratio variations with time for DPPG monolayer on different subphases was close to the reference one. Only more intense changes occurred for DPPG film on the AA/TiO_2_ and AA/HA/TiO_2_ subphases, where a greater pressure drop was noted at first 10 min. However, the quite stable level of π/π_0_ ratio was achieved in a comparable time as for DPPG layers on the remaining subphases. For these two cases, specific/characteristic hysteresis loops were also noted during the compression-decompression measurements with downtime 1000 s (Figure 2B). Although a slight downtime plateau was observed, there was a drastic drop in surface pressure as soon as the decompression cycle was initiated (the descending curve is almost perpendicular to the *x*-axis). This may suggest the intercalation of TiO_2_ particles between DPPG molecules that are able to form strong bonds with PO_4_^−^ not governed by electrostatic forces alone, but also of specific adsorption [29,30,31,32]. TiO_2_ interacts with adjacent DPPG molecules, during the entire compression cycle, on the one hand, to some extent stabilizing the monolayer. On the other hand, they make it difficult for DPPG monolayers to take the place of individual phase transitions and create a compact structure. TiO_2_ increases the monolayer fluidity, as observed in Figure 1B.

Lower stability of the DPPG monolayers (expressed as π/π_0_ = f(t), Figure 2A) in the presence of AA/TiO_2_ and AA/HA/TiO_2_ in relation to the others, resulting from the fact that DPPG molecules adsorbed on TiO_2_ surfaces, even during high compression, provide greater possibilities of the spatial arrangement of hydrophobic chains (because they were separated by TiO_2_ particles). In turn, a dramatic decrease of pressure reflected in decompression curves (Figure 2B) suggests that these interactions are so strong that DPPG molecules do not loosen during the expansion of monolayers, but the rupture of the lipid film probably takes place.

The use as a subphase of AA and AA/HA for DPPG monolayers did not reveal any specific interactions. The relative surface pressure of the DPPG films on the AA and AA/HA during 1 h did not differ from the typical changes recorded for the reference (DPPG on the water), and their thicknesses over the entire compression cycles were very similar. The compression-decompression curves were also identical, with almost no hysteresis loops. This suggests that AA and HA did not disturb the structure, packing, or stability of the DPPG monolayers.

In contrast, a special type of interaction occurred between DPPG molecules and chitosan. All DPPG films recorded on subphases containing this biopolymer (AA/Ch, AA/Ch/TiO_2_, AA/Ch/HA, AA/Ch/HA/TiO_2_), despite demonstrating deviations from the typical course of the π-A curve (Figure 1A), showed stability at an almost identical level (Figure 2A and Figure S1). Moreover, the same hysteresis loops during compression-decompression measurements were recorded for them. This indicates that the contact of DPPG with Ch creates strong interactions which at the same time enable the production of a slightly looser packing (Figure 1B) but stable monolayer. These interactions take place during the initial contact and are stable under all conditions (compression, decompression, for 1 h in a closely packed monolayer). More precisely, these interactions are so strong and dominant that it is impossible to see the influence of other substances present in the subphase (HA, TiO_2_), regardless of their combination.

#### 3.1.3. Thickness and Structure of the DPPG Monolayers

An important aspect of lipid film stability in highly compressed states is the packing of lipid molecules and the presence of ordered and disordered regions. Real-time images from the Brewster angle microscope can provide information in this respect. In addition, based on measurements of the reflectivity, the relative thickness of DPPG monolayers (Table 2) deposited at the subphase/air interface was investigated.

**Figure 3:**
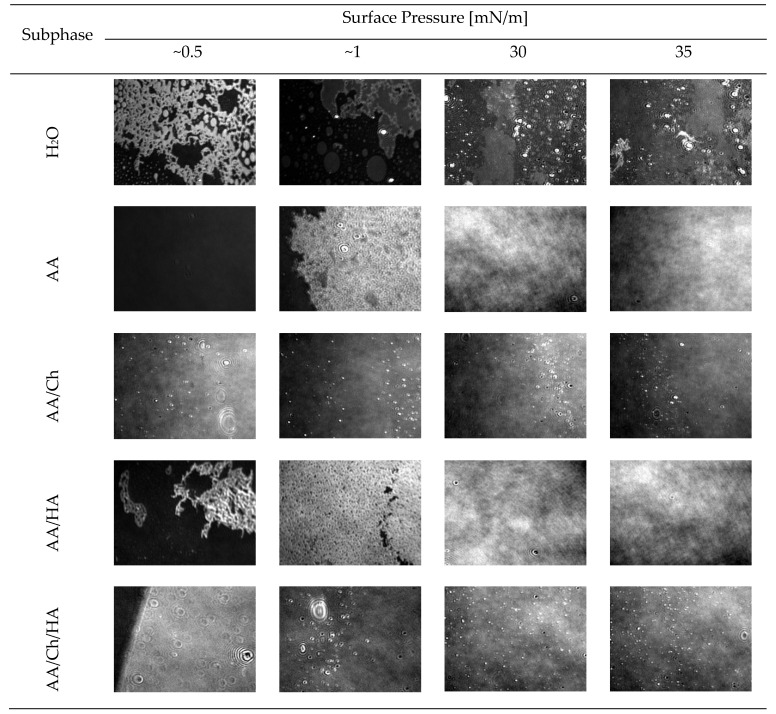
Structure of the DPPG monolayers during their compression (at surface pressures of 0.5, 1, 30 and 35 mN/m) on the different subphases (AA—acetic acid, AA/Ch—chitosan on acetic acid, AA/HA—hyaluronic acid in acetic acid, AA/Ch/HA—chitosan in acetic acid with the addition of hyaluronic acid).

The π-A isotherm (Figure 1A) and BAM images recorded for the DPPG monolayer compressed on the pure water subphase (Figure 3) are consistent with those presented in the literature [26,27,33,34,35]. Although the presence of AA and HA in the subphase did not cause significant differences in the π = f(A) and C_S_^−1^ = f(π) plots (Figure 1) for DPPG monolayers, observation with BAM (Figure 3) showed that these substances influenced the structure of the film during compression. They contributed to the creation of a more compact and homogeneous structure but did not change its thickness relative to the reference (DPPG on the water). That means the HA presence did not disturb the DPPG monolayer, but only caused an “improvement” of the compression process due to accelerating the appropriated phase transitions (from gas through expanded and condensed liquid to solid state). This may be an effect of repulsion interaction between negatively charged PO_4_^−^ headgroups of DPPG and carboxylic groups of HA.

On the other hand, the DPPG film on the AA/Ch subphase below π = 15 mN/m showed a thickness of about 1 nm smaller and above this pressure-about 1 nm greater compared to the film on H_2_O (Table 2). Moreover, as mentioned before, Ch caused an increase of DPPG monolayer fluidity. Therefore, in the BAM images, the characteristic condensed domains of DPPG that occurred for the water subphase were not observed. In addition, during compression, the grain-shaped areas were visible, whose amount accreted with increasing the surface pressure. These small domains comprise the condensed phase of DPPG monolayer. These LC phase islands appear porous, as the dark gaseous phase coexists with the bright domain structures. This suggests that Ch is probably located preferably in loose monolayer areas in the liquid expanded phase, creating Ch-rich and lipid-poor regions. Chitosan can not only interact electrostatically with charged groups, but can also produce hydrophobic interactions with the lipid tails [36]. Consequently, Ch may insert between DPPG molecules, causing the larger expansion of the examined monolayers, which was also reflected as the decrease in the monolayer packing (Figure 1B). In effect, these physical interactions between Ch molecules and phospholipids possibly account for the enhanced permeability of biological membranes induced by Ch molecules. Similar changes for the DPPG monolayer were also observed by [33] using DMSO solution as the subphase. This effect was assigned the condensing and caging effect of DMSO on the DPPG monolayers, although it is a negatively charged lipid. The observed condensing effect of DMSO molecules on phospholipids physically changes the morphology of the film, creating DMSO-rich and lipid-poor regions. Moreover, the adsorption kinetics recorded during penetration experiments carried out by [27] revealed the strongest adsorption of the Fe_3_O_4_-Ch nanoparticles in the DPPG monolayer.

Such a structure of DPPG film was also observed on the AA/Ch/HA subphase. Therefore, in the most compressed state, the expansion of the DPPG films occurs and the Ch molecules remain adsorbed into them. Here, however, the appearance of a lipid-poor region at 15 mN/m was additionally observed as a drastic decrease in monolayer thickness, which confirms the above suggestions.

### 3.2. The Influence of Ch, HA and TiO_2_ on the S. aureus Lipids Monolayer

#### 3.2.1. Behavior and Packing of *S. aureus* Lipid Monolayers during Compression

**Figure 4:**
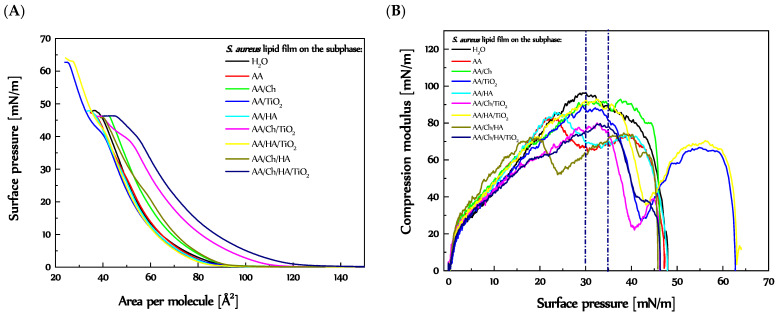
(**A**) π-A isotherms obtained by means of the Langmuir technique and determined based on them (**B**) C_S_^−1^ = f(π) relationships for *S. aureus* lipid monolayers on the individual subphases (more details Figure S2).

The π-A isotherm for the film composed of lipids isolated from *S. aureus* species registered on the H_2_O subphase was called the reference (Figure 4). The π-A isotherm plotted has a mild course without any visible inflections indicative of phase transitions. The surface pressure began to increase at an area of A_0_ = 93.9 Å^2^. The monolayer collapse occurred at π = 47.3 mN/m (Figure 4A). Used as subphases the AA and AA/HA solutions caused a slight shift of the *S. aureus* lipid isotherms to smaller areas, A_0_ = 89.6 Å^2^ and A_0_ = 88.1 Å^2^, respectively. The monolayers collapsed at almost the same surface pressure π_coll_ = 46.7 mN/m and 46.8 mN/m, respectively. The shapes of the π-A plots were maintained, although a little inflection at π~27.5 mN/m took place. This was reflected in the graph of compression modulus (C_S_^−1^) versus surface pressure. Clearly, for the *S. aureus* lipid film on the H_2_O subphase, the C_S_^−1^ values rose from the compression beginning, reaching a maximum at 30 mN/m (C_S_^−1^ = 95.2 mN/m). In turn, in the case of AA and AA/HA subphases (Figure 4B), two maxima were visible at π = 25 mN/m (C_S_^−1^ ca. 85 mN/m) and 40 mN/m (C_S_^−1^ ca. 75 mN/m), and minimum at 30 mN/m (C_S_^−1^ ca. 70 mN/m).

A different course of the π-A plot occurred for the *S. aureus* lipid film on the AA/TiO_2_ subphase. Despite a similar progression at the beginning of compression, i.e., the G-LE transition took place at A = 94.7 Å^2^ (near the reference), followed by an increase in π with tendency similar to that on the H_2_O subphase. However, two inflections were observed, the first one at π ~ 40 mN/m and the second one at π = 62.5 mN/m. The same shape of a graph was received for *S. aureus* lipid film on the AA/HA/TiO_2_. Only slight shift of the plot to smaller molecular areas took place, A_0_ = 88.7 Å^2^. In Figure 4B, two maxima of C_S_^−1^ values were observed, at π = 32.5 mN/m and 55 mN/m. The inflections at π~40 mN/m were visible as a minimum of C_S_^−1^ = f(π) in Figure 4B. That drastic decrease in packing degree may suggest the expulsion of at least one of the ingredients. For the sake of clarity, the *S. aureus* cells consist of cardiolipin (CL) in 42%, and phosphatidylglycerol (PG) in 58% [37,38]. Based on the literature data concerning the behavior of the pure CL [23,39] and PG monolayers (previous section) on different kinds of subphases, it can be concluded that the CL monolayer collapsed earlier than that of PG. The two inflections were also observed for the *S. aureus* lipid films on the AA/Ch/TiO_2_ and AA/Ch/HA/TiO_2_ subphases. Thus, in Figure 4B, for these cases, a minimum of the C_S_^−1^ = f(π) function was also visible (at π = 40 mN/m), dividing the graph into two maxima, at π ca. 32.5 mN/m and ca. 44 mN/m. A difference was the surface pressure value of collapse, π~46 mN/m, smaller even than a reference one. In addition, these isotherms (Figure 4A) were shifted to larger molecular areas, A_0_ = 113.4 Å^2^, and A_0_ = 128.5 Å^2^, respectively.

For the *S. aureus* lipid monolayer on the AA/Ch subphase, the shape of the π-A plot was maintained (relative to a reference), only shifted to slightly higher areas, A_0_ = 96.2 Å^2^, and collapsed at some lower pressure value, π_coll_ = 46.2 mN/m (Figure 4A and Figure S2). This was also reflected in the C_S_^−1^ = f(π) relationship. For lipid film on that subphase, only one maximum of the compression modulus values was noted (at π ca. 37.5 mN/m). However, in the case of the AA/Ch/HA subphase, despite the fact that the take-off area of the π-A isotherm for *S. aureus* lipid film was similar (A_0_ = 95.5 Å^2^) to that on AA/Ch, and the collapse pressure was the same, a change in the course of plot was observed. More precisely, the small inflection at π ca. 22.5 mN/m appeared (Figure 4A and Figure S2). That was visible in Figure 4B as a minimum of the graph between two maxima, at π ca. 20 mN/m and π ca. 38 mN/m.

Generally, despite all changes in the course of the plot that occurred in Figure 4B in relation to the reference, the *S. aureus* lipid films on all subphases possessed compression modulus values in the range characteristic for the liquid phase.

Interestingly, for the *S. aureus* lipid films on the H_2_O, AA/TiO_2_, and AA/HA/TiO_2_ subphases, similar values of A_lim_ parameter were noted, 64.4 Å^2^, 65.0 Å^2^, and 65.5 Å^2^, respectively. This suggests that these components of the subphase (mainly TiO_2_) did not penetrate into the *S. aureus* monolayer, but lipids molecules could adsorb on the TiO_2_ particles causing an expulsion of the CL or PG. Slightly larger A_lim_ values were received for the *S. aureus* lipid film on the AA (68.5 Å^2^) and AA/HA (66.7 Å^2^) subphases. This indicates that the acidic environment leads to the weakness of the attraction forces between lipid molecules in the Langmuir layer.

A substantial increase in the A_lim_ values occurred for the *S. aureus* lipid film on the Ch-containing subphases, namely AA/Ch (71.2 Å^2^), AA/Ch/HA (77.9 Å^2^), AA/Ch/TiO_2_ (85.6 Å^2^), and AA/Ch/HA/TiO_2_ (91.8 Å^2^). This implies that Ch can penetrate the monolayer made of *S. aureus* lipids, weakening the attraction forces between them. Moreover, a Ch-induced effect has been reinforced by further components.

#### 3.2.2. Stability of the *S. aureus* Lipid Monolayers

**Figure 5:**
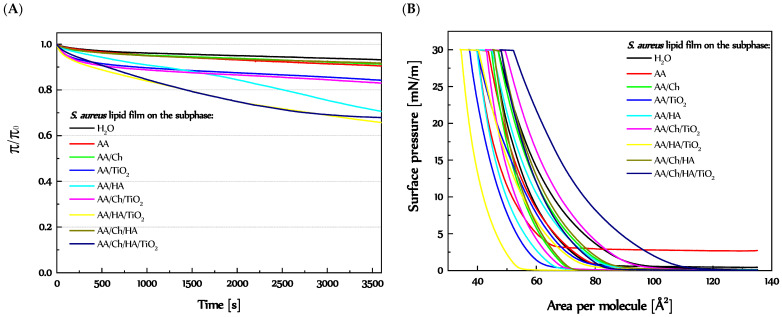
(**A**) π/π_0_ = f(t) and (**B**) hysteresis loops (more details Figures S3 and S4) obtained by compression and decompression of the *S. aureus* lipid monolayers on the individual subphases.

To analyze the changes in the structure of *S. aureus* lipid film taking place at the pressure corresponding to the pressure of biological membranes in more detail, the stability of the *S. aureus* lipid film at π = 30 mN/m over one hour and compression-decompression measurements were done (Figure 5).

The stability of *S. aureus* lipid monolayer compressed to 30 mN/m was reflected as π/π_0_ ratio versus time (Figure 5A). Reference monolayer had very stable relative pressure over one hour. A minimal decrease of π/π_0_ value was observed. Interestingly, almost the same level was reached by *S. aureus* lipid film on the AA, AA/Ch, and AA/Ch/HA subphases. In turn, for membrane on AA/HA subphases from the beginning the decrease of surface pressure was occurred, more intense after 29 min. This suggests that the interactions between Ch and lipid molecules during compression were so strong that they made it impossible of relaxation lipids molecules at the interface. On the other hand, HA did not influence a membrane during compression, but only had the ability to interact on their packed form. A slightly greater decrease of π/π_0_, at the beginning, appeared for *S. aureus* lipid film on the AA/TiO_2_ and AA/Ch/TiO_2_ subphases, in relation to the reference, but a relatively stable level was reached after just 10 min. A different situation occurred in the case of AA/HA/TiO_2_ and AA/Ch/HA/TiO_2_ subphases. The lipid films on both subphases were not stable in time. Only for *S. aureus* lipid film on AA/Ch/HA/TiO_2_ subphase was a constant π/π_0_ value reached after 45 min.

The obtained results are discussed more accurately in the next section.

The magnitude of the hysteresis loops obtained during compression-decompression measurements provides information about the existence of interactions between lipids molecules (in a closely packed layer) and subphase components, as a result of which the loss of material from interface could have occurred. Both compression and decompression curves for *S. aureus* lipid film had similar courses on each of the subphases (except AA) (Figure 5). In all cases, the descending curves were shifted to smaller molecular areas, besides reference. In effect, hysteresis loops of considerable sizes were obtained (Figures S3 and S4).

Interestingly, the decompression curve of the *S. aureus* lipid film on H_2_O was shifted to the larger areas per molecule. At the beginning of the decompression process, it was about 2 Å^2^, but total relaxation was achieved at area larger by about 12.3 Å^2^. This suggests that during compression the highly packed aggregates, separated by empty areas, could be created, which at constant surface pressure value (at downtime) relax. In turn, using the AA solution as a subphase caused a shift descending curve (at the start) about 4 Å^2^ to the left side of the graph, but total decompression occurred at π ca. 2.5 mN/m (did not achieve zero surface pressure) at area ca. 18.4 Å^2^ smaller. A slightly bigger plateau at downtime in relation to the reference on the AA/Ch, AA/TiO_2_, and AA/Ch/HA was noted (2.6 Å^2^, 2.8 Å^2^, and 2.9 Å^2^, respectively). Ultimately, in these cases, the decompression monolayer occurred at 18.3 Å^2^, 19.7 Å^2^, and 18.3 Å^2^ in the smaller areas, in reference to the compression curves. The greater hysteresis loops were obtained for *S. aureus* lipid films on the AA/Ch/TiO_2_, AA/HA/TiO_2_, and AA/Ch/HA/TiO_2_, and the differences in the G-LE phase transition area between ascending and descending curves were 28.0 Å^2^, 26.8 Å^2^, and 27.6 Å^2^, respectively (Figure S4). Meanwhile, the length of the plateau at downtime was in sequence 6.7 Å^2^, 6.3 Å^2^, and 4.6 Å^2^. In turn, a decompression curve of *S. aureus* lipid monolayer on an AA/HA subphase was shifted to the left side of the graph about 5.4 Å^2^ at 30 mN/m and 19.8 Å^2^ at π~0 mN/m (Figure S3). Taking into consideration effects involving by AA/Ch, AA/HA, and AA/TiO_2_ subphases on model *S. aureus* lipid monolayer, it could be concluded that Ch and TiO_2_ affect the monolayer structure during compression, downtime, and decompression in a constant manner. In turn, HA has a greater impact on a packed monolayer (during downtime). This is related to the charge of the individual components of the subphases. HA with a negative charge causes repulsion of the lipid molecules (also negatively charged), which is more intense at downtime. In effect, the attraction forces between lipids become stronger, creating more condense and packed regions occupying smaller areas. More considerations have been made as regards the structure and thickness of *S. aureus* lipid film (in the next section).

#### 3.2.3. Thickness and Structure of the *S. aureus* Lipid Monolayers

Taking into account that the Langmuir film is not a pure component but rather a mixture of lipids (in this case CL and PG), in order to draw more precise conclusions, it is necessary to analyze the membrane behavior under other conditions in more detail.

**Figure 6:**
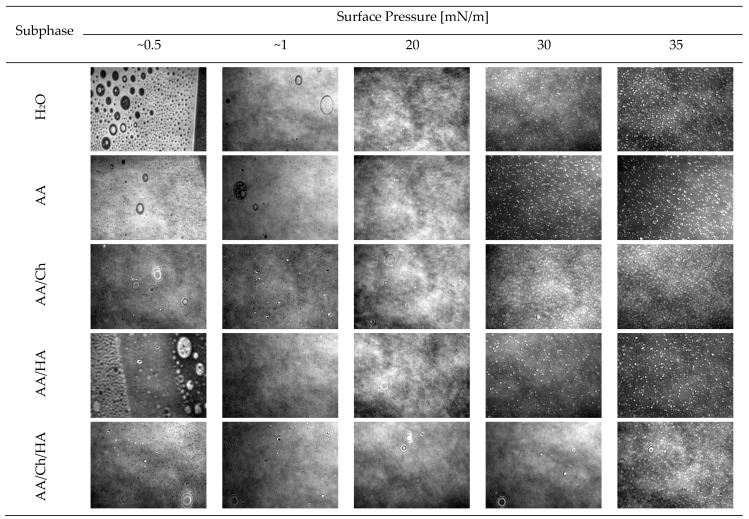
Structure of the *S. aureus* lipid monolayer during their compression (at surface pressures of 0.5, 1, 20, 30 and 35 mN/m) on the different subphases (AA—acetic acid, AA/Ch—chitosan on acetic acid, AA/HA—hyaluronic acid in acetic acid, AA/Ch/HA—chitosan in acetic acid with the addition of hyaluronic acid).

Therefore, the structure of *S. aureus* lipid films was analyzed during compression using the Brewster angle microscope, obtaining information in real-time on the shape of domains formed (Figure 6) and the thickness of the membranes (Table 3). The morphology of the monolayers obtained on individual subphases is presented in Figure 6.

The *S. aureus* lipid film on the H_2_O subphase formed condensed, bright domains even at low values of the surface pressure π > 5 mN/m. The structure of the film resembled a grid with round, symmetrical holes of various diameters. As the compression progressed, these holes decreased to form a more compact and uniform layer. However, up to π < 5 mN/m, slight defects in the form of black-colored holes were observed, indicating the existence of small regions of non-condensed state. The number of these defects decreased with the increase of π value. On the other hand, at π > 20 mN/m, domains in the form of bright points began to appear, and their amount increased with the progress of compression. The highest quantity was observed at π = 35–40 mN/m, which could indicate partial miscibility of the lipid components in the monolayer and possible ejection of those having a lower collapse surface pressure value [40]. Both π = f(A) and C_S_^−1^ = f(π) relationships plotted for this lipid film suggest that its collapse took place above surface pressure value, π > 45 mN/m. Moreover, the thickness (Table 3) of the obtained film increases, until π = 40–45 mN/m. Overall, *S. aureus* lipid film thickness during compression slowly increases with surface pressure π, from 1.4 nm for π = 0.5 mN/m to 2.7 nm for π = 40–45 mN/m.

The *S. aureus* lipid film compressed on the acetic acid subphase showed an upper level of condensation at the same pressure values compared to the *S. aureus* lipid film on the H_2_O, while the entire structure was retained. This is also confirmed by the values of the thickness of *S. aureus* lipid layer (Table 3) at the respective surface pressures, which were from 0.1 to 0.3 nm higher compared to the values obtained on H_2_O. At low surface pressure values, less condensed areas in the form of black craters were also observed. Almost no bright highly condensed fragments were present at the start of compression. On the other hand, at π > 25 mN/m, aggregates appeared in the form of white, bright dots, which remained to π = 35 mN/m, then their amount significantly decreased.

As expected, the use of a mixture of acetic and hyaluronic acid as a subphase caused a visible change in the structure of the *S. aureus* lipid film (Figure 6). The coexistence of a reticular structure with small, round holes and elliptical, bright (more condensed) areas was observed. A homogeneous structure at π < 1 mN/m was formed, but with numerous black holes/craters. As the pressure increases, at the π~4 mN/m (data not shown), threadlike bright packed structures were also observed, and bright “glowing” dots/aggregates resembling the structure of the DPPG monolayer on H_2_O subphase (Figure 3). However, above surface pressure value π > 5 mN/m, a relatively homogeneous lipid layer of *S. aureus* was obtained, without any defects. On the other hand, above π > 25 mN/m, white points began to appear, as they appeared in *S. aureus* lipid films prepared on other subphases. In this case (on the AA/HA subphase), their greatest amount occurred at π = 40 mN/m. As a result of these changes, the thickness (Table 3) of the *S. aureus* lipid monolayer was also slightly higher than the reference one (differences at the level of 0.1–0.3 nm).

In the presence of chitosan in the subphase (AA/Ch) the complete disappearance of the reticular structure of *S. aureus* lipid film was obtained (Figure 6). At the beginning of compression, a condensed layer with numerous aggregates was observed. The monolayer did not create a fully dense structure throughout the compression cycle. Two types of structure were visible at all times, the first in the form of black holes/craters, evidencing the co-existence of domains in more and less condensed phases, and the second in the form of white spots (revealed at the pressure of 10 mN/m < π < 20 mN/m), indicating the existence of highly condensed regions. In addition, specific elliptical “blinking” structures appeared, which had already been observed previously for monolayers of other phospholipids registered on chitosan-containing subphases [Ładniak et al., personal communication]. All defects occurred regardless of the prevailing π, and their amount dropped sharply above π > 5 mN/m. On the other hand, at π > 30 mN/m, bright points were revealed (also described for other subphases). The appearance of a compact (but with numerous domains) structure of the *S. aureus* lipid film was also reflected in its thickness (Table 3), which was greater than the reference one from 0.2 to 0.8 nm (taking into account the entire compression cycle).

A situation similar to that of the above-discussed monolayer on the AA/Ch subphase occurred for the lipid film on the subphase containing both biopolymers, i.e., AA/Ch/HA. In this case also the monolayer was not homogeneous, it had the same domains (Figure 6). The difference is that the structures with greater condensation were visible in significant amounts at value π > 35 mN/m. However, the thickness of the *S. aureus* lipid layer on AA/Ch/HA subphase was not as significantly different as that on the AA/Ch subphase. It was only slightly less than the reference one by 0.1–0.2 nm (Table 3).

In summary, the presence of Ch and Ch/HA in the subphase had the greatest effect on the changes of the structure of *S. aureus* lipid layer during the compression process. Simultaneously, BAM images allowed observation of the growing lipid domains (Figure 6). Their presence was evidenced by bright points appearing on the dark subphase background, which combine with increasing pressure to form a uniform film. On the other hand, bright white aggregates appearing at π~30 mN/m may also denote the existence of partial miscibility of lipid components in the monolayer. As a consequence, strong hydrogen bonds can form between the same group of film components, more specifically between neighboring lipid molecules. The uprising of these permanent interactions means that during expansion it was not possible to return them to the state before compression. Consequently, the decompress curve was shifted towards much smaller molecular surfaces, creating large hysteresis loops and a significant plateau at maximum compression pressure (during downtime) (Figure 5B), which can suggest the loss of lipid material from the interface.

The situation of the monolayer in the region at surface pressure π = 30 mN/m, and as a result of strong interactions between the components of the lipid film, causes “partial” stabilization of this state. Therefore, when recording the stability over time, no significant changes in the relative surface pressure were observed. The lack of visible relaxation of molecules in the lipid film after ending the compression process proves that the molecules have already adapted their spatial orientation to the prevailing surface conditions.

Only in the case of the *S. aureus* lipid film created on the AA/HA subphase was a sharp decrease of relative surface pressure after 0.5 h observed (Figure 5A). In this case, however, at π = 30 mN/m, a smaller amount (than in the monolayer for AA/Ch subphase) of monolayer inhomogeneities was observed (Figure 6). This can mean that under this pressure, the monolayer is more homogeneous. In turn, that suggests that HA affects the *S. aureus* lipid monolayer, but not as significantly as Ch. This is probably due to differences in the charge of both polysaccharides, HA is a polyanion with a strong negative charge and Ch is a polycation which can strongly interact with CL (one of components of the *S. aureus* monolayer with negative charge). This conclusion may be supported by the fact that CL, because of the restricted movement resulting from its size, is not able to form intra- or intermolecular hydrogen bonds that can stabilize the negative charge of the head. Therefore, the unshielded negative charge can easily interact with protons and cations in solution, e.g., chitosan molecules. The interaction reduces the effective size of the polar head, promoting the formation of nonlamellar structures [41]. In addition, CL induces the folding structure locally on the membrane, before reaching the collapsed state upon further compression [39]. That effect can be also strengthened by the Ch-presence. Consequently, we can observe the increase of the thickness and packing degree of the *S. aureus* lipid monolayer. The model for a folding structure assumes that the increased interfacial area forming a curvature helps the CL molecules to minimize the repulsive force caused by the unsaturated hydrocarbon chains and the negatively charged headgroups. However, the collapsed structure is an even more thermodynamically favorable process. In the collapsed structure, the internal repulsion between hydrocarbon chains is drastically reduced, and the negatively charged headgroups repulsion is completely gone [39].

Due to technical reasons, i.e., too much scattering of the laser light emitted by the BAM by the large TiO_2_ particles, it was impossible to record the structure and thickness of *S. aureus* lipid films on the subphases containing this component. However, taking into account the above dependencies, we can conclude that both the π/π_0_ ratio over time for *S. aureus* lipid films on AA/TiO_2_ and AA/Ch/TiO_2_ subphases and the significant size of hysteresis loops (mainly on AA/Ch/TiO_2_) also may indicate the partial miscibility of lipid components in the membrane at π = 30 mN/m or its collapse. As a result, both subphases have a destructive effect on the monolayer made of lipids isolated from *S. aureus*.

In turn, the π/π_0_ = f(t) ratio determined for the *S. aureus* lipid films on the AA/HA/TiO_2_ and AA/Ch/HA/TiO_2_ subphases decreased with time. On the one hand, this would suggest no monolayer collapse at π = 30 mN/m, so the decrease in π/π_0_ would result from the relaxation of the lipid mixture at the interface. On the other hand, taking into account the fact that after 2500 s the *S. aureus* lipid film on AA/Ch/HA/TiO_2_ reached a stable level of π/π_0_ ratio, while on AA/HA/TiO_2_ it continued to decline after analogous time, we can conclude that their effect on the membrane was not the same and each of the cases discussed must be considered separately.

Based on the results obtained for AA/Ch and AA/Ch/TiO_2_ subphases, the following changes can be visible as an effect of TiO_2_ addition to Ch during *S. aureus* lipid film formation: (I) shift of the *S. aureus* lipid film π-A isotherm towards higher molecular areas, (II) decrease in the C_S_^−1^ values, (III) lowering π/π_0_, but maintaining stability for 1 h, (IV) increasing the hysteresis loop; (in relation to data received for *S. aureus* lipid film on the AA/Ch subphase). The similar modifications of the *S. aureus* lipid film were observed (except for point II) after the introduction of TiO_2_ particles into the mixed AA/Ch/HA subphase. It is also worth noting that the *S. aureus* lipid film on the AA/Ch/HA subphase showed higher inhomogeneity, suggesting partial miscibility of lipid components, than existed on the AA/HA subphase. Considering, in the same way, the influence of TiO_2_ addition to the AA/HA subphase on the behavior of the *S. aureus* lipid film, we do not observe the above-mentioned relationships (I–IV). Thus, we can conclude that the use of the AA/Ch/HA/TiO_2_ subphase could also have had a destructive effect on *S. aureus* lipid monolayer. Moreover, the decrease in π/π_0_ could be dictated not only by lipid relaxation, but also by additional interference of such a complex subphase on the lipid components present in the membrane. On the other hand, the combination of TiO_2_ with HA could have contributed to the further reduction of the disturbance effect on the *S. aureus* lipid monolayer. As a result of the existence of competition for interactions between the negatively charged lipid components of the *S. aureus* lipid layer and HA molecules with TiO_2_ particles.

### 3.3. The Influence of the Ch, HA and TiO_2_ on the E. coli Lipids Monolayers

#### 3.3.1. Behavior and Packing of *E. coli* Lipid Monolayers during Compression

The π-A isotherm for the film composed of lipids isolated from *E. coli* registered on the H_2_O subphase was called the reference. For this case, the G-LE phase transition occurred at A = 85.5 Å^2^. As the compression progressed, the surface pressure increased, finally, the monolayer collapsed at surface pressure value π = 47.4 mN/m. The shape of the isotherm did not reveal any inflections. On the other hand, the dependence of the compression modulus (C_S_^−1^) as a function of surface pressure indicated that the monolayer was mainly present in the expanded liquid state. Only in the pressure range of 20–30 mN/m, the C_S_^−1^ had the values characteristic for condensed liquid.

The use of AA solution as a subphase resulted in a faster pressure increase in the range of π 0–25 mN/m (A_0_ = 86.2 Å^2^). On the other hand, at higher pressure values, the isotherm course for *E. coli* lipids was similar to the reference. After all, layer collapse occured at slightly lower value π = 46.3 mN/m. Moreover, the film in the entire pressure range did not exceed the C_S_^−1^ values characteristic for the state of expanded liquid. A similar relationship was observed for the *E. coli* lipid film on the subphase with hyaluronic acid (AA/HA). There was also a shift in the G-LE transition towards larger areas (A_0_ = 87.8 Å^2^). However, above the pressure of 25 mN/m, the isotherm course was the same as that on the AA subphase (Figure S5). Moreover, the plot of the C_S_^−1 =^ f (π) relationship was similar. In both cases (AA and AA/HA subphases), the maximum C_S_^−1^ values for *E. coli* lipid film were observed at 15, 25, and 40 mN/m surface pressures.

The use of TiO_2_ in the subphase (AA/TiO_2_) resulted in visible changes in the course of the isotherm for the *E. coli* lipids layer. The G-LE transition took place at A = 92.2 Å^2^. Thereafter, there was a much milder increase in pressure as the compression progressed. Additionally, an inflection was observed at a pressure of approximately 40 mN/m, which can indicate a collapse of one or more constituents of the monolayer, or their partial miscibility. In turn, the graph of the compression modulus as a function of surface pressure plotted two maxima of values at π value 25 mN/m and 53 mN/m. On the other hand, the observed minimum at 40 mN/m indicates partial miscibility and/or expulsion of one kind of the components (phosphatidylethanolamine (PE), CL, or PG) from the monolayer, rather than phase transition, because the C_S_^−1^ value does not drop to zero [40]. When such a situation takes place, in the monolayer the coexisting areas with packed and not-condensed domains can be observed. Higher surface pressure values may come to reveal that situation, which is reflected in a decrease in the value of the compression modulus. After combining HA with TiO_2_ in the subphase, the same course of the isotherm for the *E. coli* lipid film was recorded like that on the AA/TiO_2_ subphase. The difference is that it started at 79.7 Å^2^ and finally collapsed at π value ca. 61 mN/m. This was also reflected in the C_S_^−1^ = f(π) plot. Two peaks at π = 27 mN/m and 54 mN/m were also observed. However, in the π range 0–15 mN/m, the C_S_^−1^ values were similar to those obtained for *E. coli* lipid layer on the AA/HA subphase. The similar course of isotherm was received also by [42] for the POPE/POPG (3:1 ratio) on the 15 mM NaCl solution subphase. Additionally, after adding the CL to this Langmuir film, disappearance of the plateau at ~40 mN/m was noted [43]. Considering the fact that in our case *E. coli* lipid film consists of PE, PG, and CL, it can be concluded that the presence of positive charged TiO_2_ particles and further HA (containing Na^+^ ions) provides an expulsion of CL from the monolayer, simultaneously stabilizing the created membrane.

However, the use of chitosan with acetic acid as a subphase did not affect the shape of the *E. coli* lipid film isotherm compared to the reference. There was only a shift in its course towards larger areas (A_0_ = 90.5 Å^2^). A plateau suggesting a collapse took place at surface pressure value π = 46.2 mN/m. A similar relationship occurred on the subphase containing both biopolymers (AA/Ch/HA). The *E. coli* lipid film during compression plotted the similar isotherm as on AA/Ch, with its position approaching the reference isotherm (A_0_ = 88.6 Å^2^). The values of the compression modulus for lipid films on these subphases (AA/Ch and AA/Ch/HA) were also practically comparable in the entire pressure range (Figure S5). The only difference was that in the AA/Ch subphase three maxima of C_S_^−1^ values were observed (at π ca. 20, 30, and 37 mN/m), but on the AA/Ch/HA subphase only two maxima (at π = 24 and 37 mN/m).

After applying a mixed subphase of TiO_2_ with chitosan, despite the fact that the isotherm for the lipid film showed a similar inflection in the region of π = 40 mN/m (as for AA/HA and AA/HA/TiO_2_), a shift of its course towards larger areas occurred (A_0_ = 98.1 Å^2^). Additionally, a much smoother increase of surface pressure π was noted when condensed domains were formed in the monolayer. Above π = 25 mN/m, the course of the curve was almost identical to that obtained on H_2_O subphase. Finally, at π = 46.7 mN/m, a plateau was visible, suggesting a collapse of the membrane. However, significant differences were noticed for the value of the compression modulus at individual stages of compression process. At the beginning of the compression, an increase in the C_S_^−1^ value was observed with a tendency comparable to that obtained for *E. coli* lipid film on AA/TiO_2_. In the range of π = 10–20 mN/m, a relatively constant C_S_^−1^ value was obtained, followed by a sharp increase, reaching a maximum at π = 35 mN/m. Then, at π = 40 mN/m, the minimum C_S_^−1^ values suggested partial miscibility and/or an ejection of some components (CL, PE, or PG) from the monolayer, as there was another increase in the modulus value, reaching a maximum at π ca. 43 mN/m. Finally, the monolayer was collapsed at π = 46.2 mN/m.

In turn, the HA-addition to the AA/Ch/TiO_2_ subphase (i.e., AA/Ch/HA/TiO_2_) contributed to the softening of the inflection at π = 40 mN/m. Moreover, the increase of π occurred at A_0_ = 93.3 Å^2^ (G-LE phase transition). Next, π values in the monolayer kept rising steadily as the compression progressed until it reached a plateau at π = 46.4 mN/m. Similarly, C_S_^−1^ initially increased with the trend also plotted by the *E. coli* lipid film on the AA/Ch/TiO_2_ subphase, but above π > 15 mN/m, the increase was slightly slower, until to a maximum of about π = 35 mN/m.

The different course of the plots for *E. coli* lipid monolayers on the AA/TiO_2_ and AA/HA/TiO_2_ subphases was caused by the enhancement of the attracting interactions between the Langmuir film components (in the case of *E. coli*, these are: CL, PG, PE), which may be result of repulsion between lipid molecules and subphase components, or the penetration of TiO_2_ particles and/or HA molecules into the monolayer (A_lim_ = 65.5 Å^2^, A_lim_ = 63.4 Å^2^, respectively). In the remaining subphases, the lipid molecules in the films occupied a larger area in a tightly packed monolayer (for AA A_lim_ = 69.8 Å^2^, for AA/Ch/HA A_lim_ = 71.3 Å^2^, for AA/HA A_lim_ = 71.7 Å^2^, for AA/Ch A_lim_ = 72.9 Å^2^, for AA/Ch/HA/TiO_2_ A_lim_ = 73.1 Å^2^, for AA/Ch/TiO_2_ A_lim_ = 76.9 Å^2^). The obtained packing parameters can prove the weakening of the attractive interactions/forces between the membrane components caused by the presence of the subphase ingredients. Moreover, it can be seen that the presence of HA in the subphase, compared to the corresponding subphase without this biopolymer, shifts the isotherm for *E. coli* lipid film towards smaller areas per molecule. Such a relationship was also observed previously when examining the effect of HA, Ch and TiO_2_ on the DPPC membrane (except AA/Ch/HA/TiO_2_) [28]. On the other hand, during an investigation of the effect of the same substances on the DOPC monolayer behaviour, a completely opposite relationship was obtained [Ładniak et al., personal communication], i.e., an increase in the A_lim_ parameter on the subphases containing HA compared to those without it.

Moreover, the observed changes resulting from the mixing in the subphase, i.e., Ch with TiO_2_ or HA with TiO_2_, in relation to differences caused by individual components, confirm our previous reports [18,28,44,45,46,47]. Clearly, in the case of the AA/HA/TiO_2_ subphase, TiO_2_ dominates in the interactions with Langmuir film components. This is completely different behaviour for AA/Ch/TiO_2_, where Ch limits/eliminates changes caused by TiO_2_. This can suggest that TiO_2_ particles are dispersed in the HA matrix in a way that did not limit their contact with the external environment. On the other hand, the Ch molecules adsorb on the surface of TiO_2_ particles, preventing their interaction with the environment. As was mentioned earlier, the differences for these polysaccharides are due to the presence of opposite charges in their molecules.

**Figure 7:**
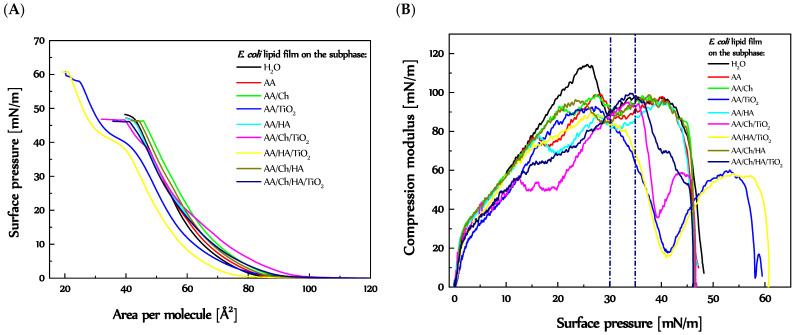
(**A**) π-A isotherms obtained by means of the Langmuir technique and determined based on them (**B**) C_S_^−1^ = f(π) relationship for *E. coli* lipid monolayers on the individual subphases (more details Figure S5).

Larger discrepancies in the position of isotherms for *S. aureus* lipid films (Figure 4) than *E. coli* (Figure 7) were an effect of the higher sensibility of PG affecting the ion presence in the subphases (caused an expansion of the isotherm) than PE. Both phospholipids (PG and PE) have the capability to form hydrogen bonds. However, in the case of PG molecules, these interactions are weaker due to electrostatic repulsions existing between negatively charged phosphatidylglycerol moieties [42].

#### 3.3.2. Stability of the *E. coli* Lipid Monolayers

**Figure 8:**
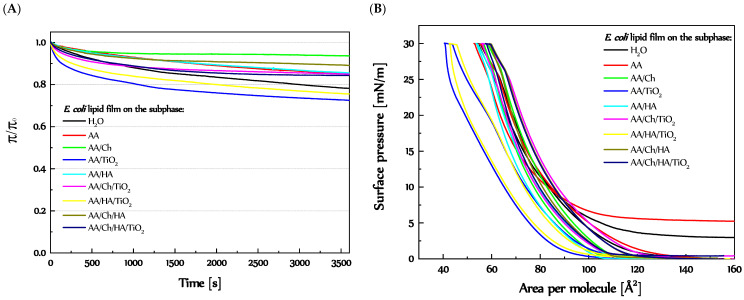
(**A**) π/π_0_ = f(t) and (**B**) hysteresis loops (more details Figures S6 and S7) obtained by compression and decompression of the *E. coli* lipid monolayers on the individual subphases.

Figure 8A presents the one-hour relative stability of the *E. coli* lipid films compressed to π = 30 mN/m on the different subphases. The most interesting fact is that the *E. coli* lipid films on almost all subphases (except AA/TiO_2_ and AA/HA/TiO_2_) achieved constant values of surface pressure the faster, than reference one. Contrariwise, for *E. coli* lipid monolayers on AA/TiO_2_ and AA/HA/TiO_2_, a greater decrease in surface pressure values occurred, although after 20 min a milder drop was observed. On the other hand, in the case of H_2_O, AA, and AA/HA subphases, a uniform decrease of π value took place all the time. In turn, *E. coli* lipid film on the other subphases got a permanent value of fewer than 20 min.

The data from compression-decompression measurements are presented in Figure 8B. For all samples, the descending curves were shifted to the left side of the graph (except *E. coli* lipid films on the H_2_O and AA subphases). The lipids film on H_2_O and AA subphases did not return to the starting state, i.e., stable surface pressure suggesting transition monolayer to the gas phase occurred at ca.3 and 5 mN/m, respectively. In addition, a downtime plateau was 1.1 Å^2^ and 5.0 Å^2^, accordingly. The *E. coli* lipid films on the AA/HA, AA/TiO_2_, and AA/HA/TiO_2_ subphases revert to the state before compression, but the descending curves were shifted at 30 mN/m about 0.7 Å^2^, 3.4 Å^2^, and 3.2 Å^2^, at near-zero surface pressure about 6.0 Å^2^, 13.2 Å^2^, and 6.0 Å^2^, respectively (Figures S6 and S7). The biggest hysteresis loop was observed for *E. coli* lipid film on the AA/TiO_2_ subphase, where the start of decompression occurred at 3.4 Å^2^ smaller area and finished at 13.2 Å^2^ smaller area, in relation to the compression curve as an effect of TiO_2_ particles.

None of the *E. coli* lipid films on the Ch-containing subphases achieved zero surface pressure after decompression, although the inflection informing about the transitions of the monolayers to the gas phase was visible, but at slightly higher π values (below 1 mN/m). Additionally, the differences in the areas of the compression and decompression curves at π = 30 mN/m and 0 mN/m were as follows: AA/Ch: 2.1 Å^2^ and 3.0 Å^2^, AA/Ch/TiO_2_: 3.4 Å^2^ and 2.8 Å^2^, AA/Ch/HA: 0.9 Å^2^ and 8.2 Å^2^, AA/Ch/HA/TiO_2_: 2.1 Å^2^ and 8.5 Å^2^ (Figures S6 and S7).

Besides, analyzing the changes of structure and stability for both lipid films (*E. coli* and *S. aureus*) on different subphases in relation to the composition of lipid layers (*E. coli* 5% CL, 15% PG and 80% PE or *S. aureus* 42% CL and 58% PG [37,38]), it can be suggested that the monolayer plateau occurring at ~40 mN/m refers mainly to CL. It is a component of both lipid films, but in various proportions. However, it is clearly visible regarding compression-decompression measurements that the size of downtime plateau closely correlates with CL amount in the appropriate bacterial lipid film.

#### 3.3.3. Thickness and Structure of the *E. coli* Lipid Monolayers

**Figure 9:**
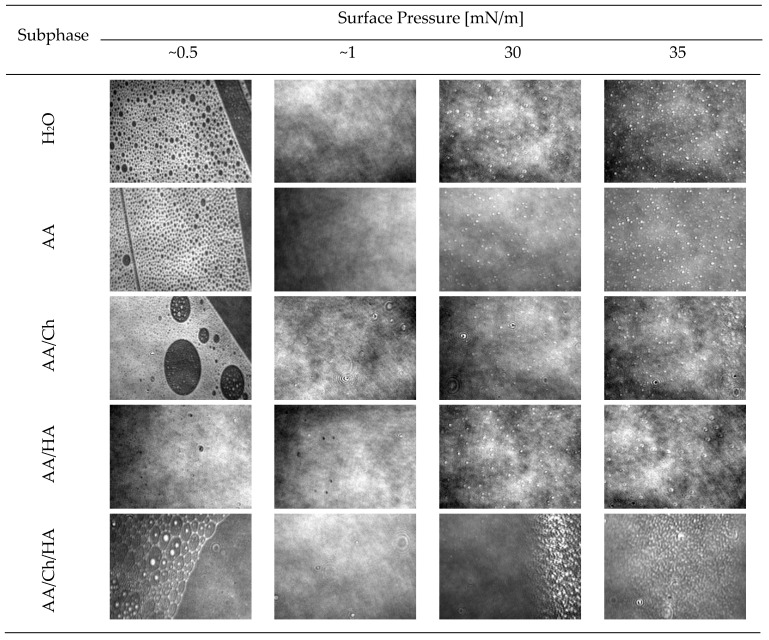
Structure of the *E. coli* lipid monolayer during their compression (at surface pressures of 0.5, 1, 30 and 35 mN/m) on the different subphases (AA—acetic acid, AA/Ch—chitosan on acetic acid, AA/HA—hyaluronic acid in acetic acid, AA/Ch/HA—chitosan in acetic acid with the addition of hyaluronic acid).

Visualization of the *E. coli* lipid film (Figure 9) on the H_2_O subphase showed that at low π values < 1 mN/m, condensed domains were formed, and the entire structure was in the form of a network with holes of various diameters. Additionally, the high-packing areas were separated by empty bands/spaces. In the π range of 1–10 mN/m, a compact, homogeneous structure with no visible defects was formed. Above π = 10 mN/m, aggregates appeared in the form of brighter points, and their amount increased with the progress of compression process. Their greatest quantity occurred at surface pressure values π > 45 mN/m. This is also reflected in the thickness of the film (Table 4), which at π = 0.5 mN/m was 1.5 nm and gradually increased to the value of 2.5 nm (π ≥ 40 mN/m).

The use of acetic acid as a subphase for the *E. coli* lipid film had no significant effect on its structure and thickness. Both features were comparable to that obtained with H_2_O subphase. Moreover, at a pressure near 1 mN/m, a compact, uniform layer was formed with visible domains above π > 10 mN/m. However, at value π > 40 mN/m a characteristic band of focused bright points was observed.

The presence of chitosan in the subphase (AA/Ch) slightly changed the structure of the obtained *E. coli* lipid film. It caused additional less condensed regions within the packed mesh structure. Moreover, it was observed that the regions with a tight-knit (reticulated) structure were more densely packed than those obtained on H_2_O and AA subphases. Ultimately, however, a compact and homogeneous layer was obtained with no visible damage/disturbance. Defects in the form of small dark craters/holes began to appear at π > 15 mN/m. At larger surface pressure value π > 25 mN/m, bright points were also observed (similar to that occurred in other cases). The greatest amount of them was found at π > 35 mN/m. However, the observed changes did not significantly alter the thickness of the *E. coli* lipid film compared to that obtained with H_2_O subphase.

Also, the mixing of acetic acid with hyaluronic acid in subphase (AA/HA) contributed to the acceleration of the formation of a compact structure of the *E. coli* lipid film, but with twice smaller thickness. At first, a meshwork structure with small holes was observed which quickly formed a uniform layer. However, at π > 20 mN/m, domains in the form of bright points gradually appeared, and their amounts increased significantly above π > 40 mN/m. The changes caused by the presence of HA in the subphase also influenced the formation of an *E. coli* lipid film of much lower thickness during the entire compression cycle, compared to all tested samples (0.4–0.5 nm in the range of 5 ≤ π ≤ 45 mN/m, relative to the reference).

The mixing of biopolymers in the subphase (AA/Ch/HA) also influenced the structure of the *E. coli* lipid film. At low-pressure values, foam-like structures were visible, which confirm the coexistence of the gas and liquid phases. However, a uniform, dense layer was quickly formed, with small structures in both forms, e.g., black holes and bright spots (seen in other cases). Additionally, above surface pressure of 25 mN/m, clusters of bright points in the form of band-zones were observed (which also occurred in the *E. coli* lipid film on the AA subphase). Despite the occurrence of these above structures, the *E. coli* lipid film on AA/Ch/HA had the greatest thickness among all the tested films (above π = 10 mN/m it had a value greater by about 0.4 nm in relation to the *E. coli* lipid film on H_2_O subphase).

Compared to BAM images obtained on the AA, AA/Ch, AA/HA, and AA/Ch/HA subphases for *S. aureus* lipid films (Figure 6), the faster appearance of domains was observed for *E. coli* films on the same subphases. As already mentioned, this was an effect of higher sensibility of PG affecting the ion presence in the subphases than PE [42]. On the other hand, Sennato et al. [48] suggest that the increase of DPPE concentration promotes the phase separation of the film into components with a different degree of stability, in addition the less stable one collapses very close to the CL collapse pressure. Thus, in this case, i.e., *E. coli* lipid monolayers, because they consist in 80% of PE, more discrepancies in the course of the π-A and C_S_^−1^ = f(π) relationships were observed.

Moreover, the use of the HA subphase (AA/HA) had a greater impact on the thickness of the resulting *E. coli* (Figure 9) than *S. aureus* lipid film (Figure 6). The structure of lipid monolayer of *S. aureus* (Figure 6) was most influenced by Ch. A consequence of adding the cationic antimicrobial agent (e.g., Ch) interacting with anionic lipids (PG, CL) could result in the emerging complex membrane domains becoming unstable and/or having altered properties. If these natural membrane domains are biologically relevant, modifying their properties may also lead to cell death [38]. In turn, the carboxylic (-COO^−^), hydroxyl (-OH), and amine groups (-NH_3_^+^) of HA can strongly interact with positively charged ethanolamine and negatively charged phosphorus groups of PE.

### 3.4. Microbiological Tests

**Figure 10:**
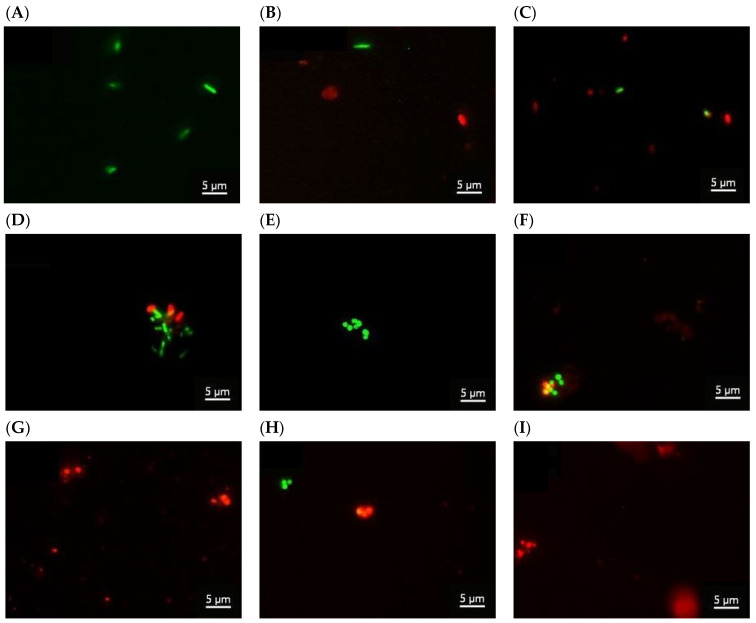
*E. coli* with Live/Dead staining and imaged with LSCM. The green signal is due to the dye SYTO9, indicating alive cells while the red signal is due to PI which marks the dead cells. (**A**) *E. coli* control, (**B**) *E. coli* treated with AA/TiO_2_, (**C**) *E. coli* treated with AA/Ch, (**D**) *E. coli* treated with AA/Ch/HA/TiO_2._ *S. aureus* with Live/Dead staining and imaged with LSCM. (**E**) *S. aureus* control, (**F**) *S. aureus* treated with AA/TiO_2,_ (**G**) *S. aureus* treated with AA/Ch, (**H**) *S. aureus* treated with AA/HA, (**I**) *S. aureus* treated with AA/Ch/TiO_2_. Magnification 1000×.

*E. coli*, a Gram-negative organism model, and *S. aureus*, a Gram-positive one, were used to study the antimicrobial activity of AA/Ch, AA/TiO_2_, AA/HA, AA/Ch/TiO_2_, AA/HA/TiO_2_, AA/Ch/HA, and AA/Ch/HA/TiO_2_ mixtures. All tested compounds showed antimicrobial activity against *E. coli* and *S. aureus* to a greater or lesser extent. However, there were considerable differences in the sensitivity to the compounds between these bacteria and methods used. The sensitivity of *E. coli* and *S. aureus* to the compounds is presented in Table 5 and Figure 10. The highest anti *E. coli* effect was caused by AA/Ch and AA/Ch/HA/TiO_2_ dispersions. The AA/Ch dispersion was responsible for the 96% mortality rate in *E. coli* in the CFU counting test and 74% in Live/Dead staining. The AA/Ch/HA/TiO_2_ dispersion decreased the viability of *E. coli* by 93% in the CFU counting test and 91% in Live/Dead staining. AA/HA/TiO_2_, AA/Ch/HA, and AA/Ch/TiO_2_ were also highly active against *E. coli*. Among the tested mixtures, AA/TiO_2_ and AA/HA showed the lowest activity against *E. coli* in both tests used.

Similar to *E. coli*, chitosan was also very active against *S. aureus*, as the killing effect was 96% in the CFU counting test and 72% in Live/Dead staining. AA/TiO_2_ was the second most lethal suspension against *S. aureus*. Although chitosan and TiO_2_ alone were the most active against *S. aureus,* 96% and 70% (72% and 65% in Live/Dead staining), respectively, no synergistic effect of their antibacterial activity in mixed sample AA/Ch/TiO_2_ was observed.

On the other hand, AA/HA was characterized by the lowest killing activity against *S. aureus*, amounting to 25% in the CFU counting assay and 35% in Live/Dead staining. Despite its high activity against *E. coli*, AA/HA/TiO_2_ showed no bactericidal activity against *S. aureus* (0%) in the CFU counting assay. However, Live/Dead staining showed the lethal effect of AA/HA/TiO_2_ on *S. aureus* (52% mortality). Probably, these differences are due to the fact that AA/HA/TiO_2_ causes *S. aureus* cells to enter a dormancy state termed viable but non-culturable (VBNC).

Comparing the effect of AA/Ch, AA/HA, and AA/TiO_2_ on the mortality of both bacteria, it is clearly visible (and simultaneously confirmed) that AA/Ch has the biggest antibacterial activity. Nonetheless, the synergism/strengthening of that property was observed for the mixture of AA/HA/TiO_2_ and AA/Ch/HA/TiO_2_ against *E. coli* in Live/Dead staining measurements. Taking into the consideration that Ch and HA possess a similar structure to the LPS in OM of Gram-negative bacteria (consisting of repeating oligosaccharide units of various sugar residues connecting by glycosidic linkages), it probably facilitates the interactions of biopolymers with the bacterial membranes. Then, the TiO_2_ particles surrounded by polysaccharides being in contact with bacteria can be successively released. In turn, the combination of Ch with TiO_2_, does not affect the *E. coli* mortality (for both AA/Ch and AA/Ch/TiO_2_ mixtures the same level of antibacterial activity is noted).

The rate of bacterial mortality under the influence of the tested compounds correlates with the values of physicochemical parameters obtained for model lipid bacterial membranes using the Langmuir technique and Brewster angle microscopy. As we expect, given that Ch has the most antibacterial activity, the greatest impact on the change of A_0_, and A_lim_ parameters, hysteresis loops, thickness, and a conformation of molecules in monolayers (Figure 6 and Figure 9) is observed for this biopolymer. In the case of Ch-containing subphases, the largest packing disturbance is manifested in the larger area occupied by molecule in the compact layer (A_lim_ was in the range of 6.8–27.4 Å^2^ for *S. aureus* lipid films, and 3.5–9.1 Å^2^ for *E. coli* lipid films) in relation to the reference lipid monolayer on water. Additionally, a strong interaction between subphase components (mainly Ch) and lipid molecules in the monolayer during compression makes the relaxation of lipid molecules at the interface difficult (Figure 5A and Figure 8A). Besides, Ch (alone or in combination with HA) has the most influence on the monolayer structures, leading to the appearance of a large amount of condensed domains (Figure 6 and Figure 9), and causing the significant increase (average of about 0.4nm) of their thickness (Table 3 and Table 4) in comparison to the reference. Despite the fact that microbiological tests did not reveal the synergism of antibacterial properties of Ch in combination with TiO_2_ and/or HA against *S. aureus*, in the Langmuir measurements an enhancement of this effect was observed. For *S. aureus* monolayers on the AA/Ch/TiO_2_, AA/Ch/HA, and AA/Ch/HA/TiO_2_, bigger A_lim_ values and hysteresis loops in relation to the bacterial layer on the AA/Ch subphase were noted. This may be because the sensitivity of the two methods is different. As far as the application aspect is concerned, it is important, because even subtle changes in the typical bacterial monolayer may, in the long run, suggest the intensification of the antibacterial properties of the tested phase.

However, considering the mortality rate of the bacteria in the microbiological tests, we have to take into account that the bacterial membranes consist of not only lipids, but also of other components, such as proteins and polysaccharides. This fact and knowledge about the structure of cell wall Gram(−) (containing IM and OM [1]) and Gram(+) (having a thick murein layer) bacteria allow to explain the different levels of bacterial mortality. Between Ch, HA, and TiO_2_ compounds and bacterial components, other specific interactions could also occur. For example, researchers [49] suggest that HA, as a component of the mammalian extracellular matrix, is used by pathogens as a carbon source during tissue invasion and replication, which may preferentially attract bacteria to the nanogel surface. Therefore, it can be an effective coating for antibacterial substances, which, as a result of “digesting” HA, can successively be released and interact with phospholipids of the bacterial membrane, increasing its permeability and causing subsequent disruption of the cell wall. Antibacterial ability was also observed by Makvandi et al. [50], who did not show the antibacterial properties of HA against Gram(+) *S. aureus*, *Bacillus subtilis,* and Gram(−) *Pseudomonas aeruginosa*, *E. coli*, but introduced Ag particles into the coatings. In turn, Abdelrahman et al. [51] observed the antibacterial effect only after the combination of HA with Ch. Similarly, Chen et al. [52] made a similar report for HA adduct obtained by N-halogenation reaction of HA.

## 4. Conclusions

The presence of Ch, TiO_2_, and HA in the subphases influenced the DPPG membrane structure, mainly by the modifications of packing degree. The greatest changes of compression modulus and average surface area per molecule occurred in the presence of Ch and/or TiO_2_ at pressures values close to those corresponding to the biological membranes. At this pressure, the DPPG monolayers formed a less condensed film on all tested subphases, compared to standard layer occurring on water. The Ch and TiO_2_ caused significant deviations from the typical behaviour of DPPG membrane in all conducted experiments. The Ch penetration caused the change in orientation of the DPPG molecules and slowed the creation of condensed domains in the monolayers. The Ch was located preferably into loose monolayers creating the Ch-rich and lipid-poor regions, producing hydrophobic interactions with the lipid tails. As an effect, increasing repulsive Coulombic forces between negatively charged DPPG headgroups occurred. In contrast, TiO_2_ particles could be located just below the surface of the lipid film and form strong bonds with PO_4_^−^ governed not only by electrostatic forces alone, but also of specific adsorption. This indirectly showed that one of the mechanisms of antibacterial action of mixtures containing chitosan, TiO_2_, and/or hyaluronic acid is probably based on bacterial membrane disturbance.

The monolayer morphology of all bacterial membranes showed inhomogeneities strictly dependent on the specific subphases compositions and in the case of *E. coli* and *S. aureus* lipid films suggested the expulsion of one of the components (CL, PE, or PG) from the monolayer or partial miscibility.

All components of the subphases influenced the structure of *E. coli* and *S. aureus* lipids monolayer but through different mechanisms. Analyzing the hysteresis loops on model *S. aureus* monolayer, it could be concluded that Ch and TiO_2_ affected the monolayer structure during compression. In turn, HA had a greater impact on a packed monolayer. This correlated to the different compositions of lipid membranes. The carboxylic, hydroxyl, and amine groups of HA could strongly interact with a positive charge of ethanolamine and an anionic charge of phosphorus groups of PE. Therefore, the use of the HA had a stronger influence on the thickness of the *E. coli* (80% PE) than on *S. aureus* lipid film (0% PE). The structure of *S. aureus* monolayer was most influenced by Ch molecules interacting with anionic lipids (PG, CL), which could result in the emerging complex membrane domains becoming unstable and/or having altered properties. In effect, these interactions between Ch molecules and phospholipids enhanced the permeability of biological membranes and may be one of the mechanisms leading to cell death of the bacteria as shown in microbiological tests.

The obtained materials with in-depth physicochemical characteristics brought us closer to functional systems useful in the production of biocompatible antibacterial films and/or skin substitutes.

## Figures and Tables

**Table 1:** Lift-off area (A_0_) and limit area (A_lim_) determined from the π-A isotherms for the DPPG monolayer on individual subphases.

Subphase	DPPG Monolayer on Different Subphases	
	A_0_ [Å^2^]	A_lim_ [Å^2^]
**H_2_O**	52.5	48.3
**AA**	51.9	46.5
**AA/Ch**	107.2	63.5
**AA/TiO_2_**	72.8	54.3
**AA/HA**	51.0	47.9
**AA/Ch/TiO_2_**	117.0	67.1
**AA/HA/TiO_2_**	68.2	50.9
**AA/Ch/HA**	110.5	65.3
**AA/Ch/HA/TiO_2_**	116.4	64.5

**Table 2:** Thickness of the DPPG monolayers during compression.

Surface Pressure [mN/m]	Thickness of DPPG Monolayer Obtained on Different Subphases [nm]				
	H_2_O	AA	AA/Ch	AA/HA	AA/Ch/HA
**0.5**	0.3	2.1	0.9	-	1.1
**5**	2.5	2.7	1.5	2.6	1.6
**10**	2.6	2.7	1.4	2.7	1.9
**15**	2.7	2.8	2.7	2.8	0.9
**20**	2.7	2.8	3.5	2.8	1.8
**25**	2.8	2.8	3.7	2.8	3.6
**30**	2.8	2.8	3.8	2.8	3.9
**35**	2.8	2.8	3.8	2.9	4.0
**40**	2.8	2.9	3.9	2.8	4.0
**45**	2.8	2.9	3.9	2.9	4.1
**50**	2.8	2.9	3.9	2.9	4.1
**55**	2.8	2.9	3.9	2.9	4.1
**60**			3.8		4.0
**65**			3.5		3.6

**Table 3:** Thickness of the *S. aureus* lipid monolayers during compression.

Surface Pressure [mN/m]	Thickness of *S. aureus* Lipid Monolayer Obtained on Different Subphases [nm]				
	H_2_O	AA	AA/Ch	AA/HA	AA/Ch/HA
**0.5**	1.4	1.7	1.7	1.5	1.6
**5**	1.8	1.9	2.0	1.9	1.8
**10**	2.0	2.2	2.0	2.1	2.0
**15**	2.1	2.3	2.5	2.3	2.2
**20**	2.2	2.4	2.6	2.4	2.3
**25**	2.3	2.6	3.1	2.5	2.5
**30**	2.4	2.7	2.8	2.6	2.5
**35**	2.5	2.8	3.0	2.8	2.7
**40**	2.7	2.8	3.0	2.8	2.8
**45**	2.7	2.7	3.1	2.7	2.8

**Table 4:** Thickness of the *E. coli* lipid monolayers during compression.

Surface Pressure [mN/m]	Thickness of *E. coli* Monolayer Obtained on Different Subphases [nm]				
	H_2_O	AA	AA/Ch	AA/HA	AA/Ch/HA
**0.5**	1.5	1.4	1.4	0.7	1.6
**5**	1.7	1.7	1.7	1.2	1.7
**10**	1.9	1.8	1.9	1.4	1.7
**15**	2.0	2.0	2.0	1.6	2.4
**20**	2.1	2.1	2.2	1.7	2.5
**25**	2.2	2.2	2.3	1.8	2.5
**30**	2.3	2.3	2.3	1.8	2.7
**35**	2.4	2.3	2.4	1.9	2.8
**40**	2.5	2.4	2.5	2.1	2.9
**45**	2.5	2.5	2.6	2.1	2.9

**Table 5:** Mortality (%) of *S. aureus* and *E.* *coli* in the presence of the compounds measured using a colony-counting assay (A) and live/dead staining with fluorescent intensity measurements (B).

Sample	Mortality (%)			
	*E. coli*		*S. aureus*	
	A	B	A	B
**AA/Ch**	96 ± 2	74 ± 6	96 ± 1	72 ± 7
**AA/TiO_2_**	78 ± 4	58 ± 7	70 ± 5	65 ± 3
**AA/HA**	73 ± 5	49 ± 4	25± 4	35±5
**AA/Ch/TiO_2_**	92 ± 2	73 ± 8	43 ± 1	41 ± 2
**AA/HA/TiO_2_**	94 ± 2	74 ± 6	0	52 ± 2
**AA/Ch/HA**	92 ± 1	82 ± 5	62 ± 1	60 ± 4
**AA/Ch/HA/TiO_2_**	93 ± 2	91 ± 1	42 ± 4	41 ± 1

## Supplementary Materials

The following supporting information can be downloaded online, Figure S1: π/π0 = f(t) relationships for the DPPG monolayer on biopolymers-containing subphases (inset: for TiO_2_-containing subphases); Figure S2: π-A isotherms obtained by means of the Langmuir technique and determined based on them C_S_^−1^ = f(π) relationships (inset) for the *S. aureus* lipid film on biopolymers-containing subphases; Figure S3: Hysteresis loops obtained by compression and decompression of the *S. aureus* lipid film on biopolymers-containing subphases; Figure S4: Hysteresis loops obtained by compression and decompression of the *S. aureus* lipid film on TiO_2_-containing subphases; Figure S5: π-A isotherms obtained by means of the Langmuir technique and determined based on them C_S_^−^^1^ = f(π) relationships (inset) for the *E. coli* lipid film on biopolymers-containing subphases; Figure S6. Hysteresis loops obtained by compression and decompression of the *E. coli* lipid film on biopolymers-containing subphases; Figure S7. Hysteresis loops obtained by compression and decompression of the *E. coli* lipid film on TiO_2_-containing subphases.

## Sample Availability

*E. coli* and *S. aureus* bacteria are available from the authors.
