# Supplementary material for: The Influence of Polysaccharides/TiO2 on the Model Membranes of Dipalmitoylphosphatidylglycerol and Bacterial Lipids
Source: Molecules. 2022 Jan 6;27(2):343. doi: 10.3390/molecules27020343 (PMC8778854; doi:10.3390/molecules27020343)
Supplement: Supplementary file 1 [file molecules-27-00343-s001.zip › molecules-1486208-supplementary.pdf]

SUPPLEMENTARY MATERIALS

The Influence of Polysaccharides/TiO_2 on the Model Membranes of Dipalmitoylphosphatidylglycerol and Bacterial Lipids

Agata Ładniak [1,2,*], Małgorzata Jurak [1], Marta Palusińska-Szysz [3] and Agnieszka Ewa Wiącek [1]

1 Department of Interfacial Phenomena, Institute of Chemical Sciences, Faculty of Chemistry, Maria Curie-Skłodowska University, M. Curie-Skłodowska Sq. 3, 20-031 Lublin, Poland, e-mail: *agata.gozdecka@poczta.umcs.lublin.pl; malgorzata.jurak@poczta.umcs.lublin.pl; a.wiacek@poczta.umcs.lublin.pl

[2] Centre for Interdisciplinary Research, Laboratory of X-Ray Optics, Faculty of Science and Health, The John Paul II Catholic University of Lublin, Konstantynów 1J, 20-708 Lublin, Poland

[3] Department of Genetics and Microbiology, Institute of Biological Sciences, Faculty of Biology and Biotechnology, Maria Curie-Skłodowska University, Akademicka 19, 20-033 Lublin, Poland, e-mail: marta.palusinska-szysz@mail.umcs.pl

* Correspondence: author: Agata Ładniak – agata.gozdecka@poczta.umcs.lublin.pl;

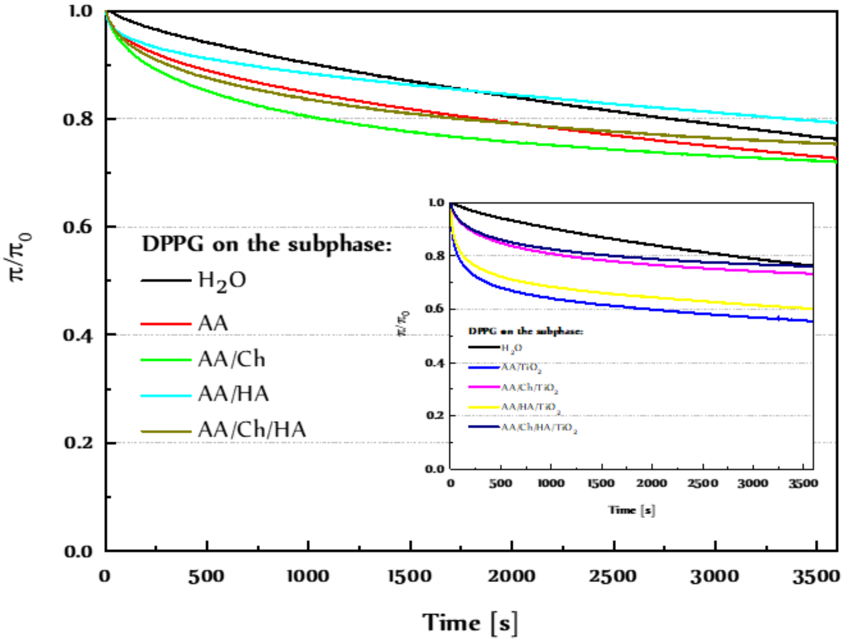

Figure S1. π/π_0=f(t) relationships for the DPPG monolayer on biopolymers-containing subphases (inset: for TiO_2-containing subphases).

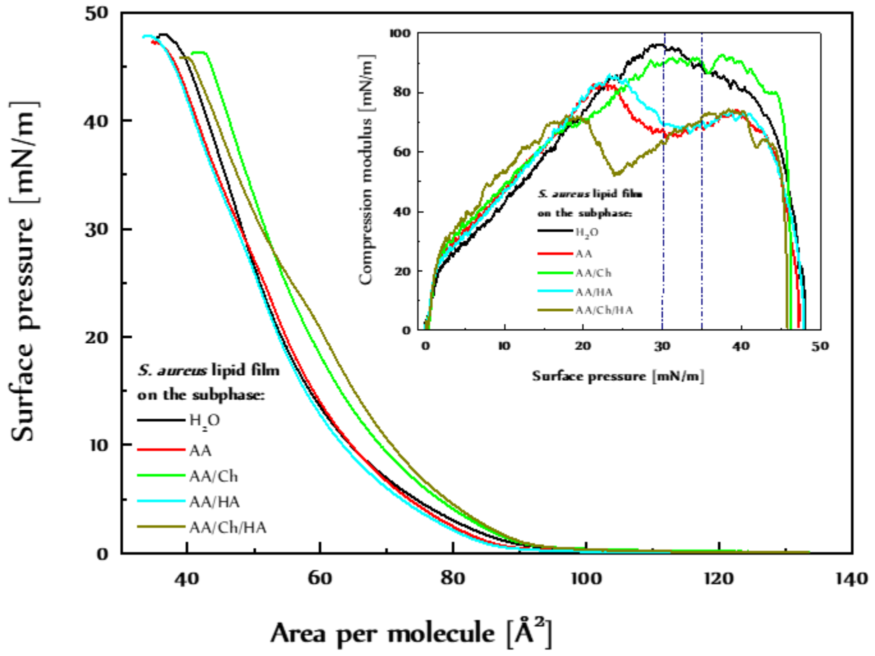

Figure S2. π-A isotherms obtained by means of the Langmuir technique and determined based on them $C_S^{-1} = f(\pi)$ relationships (inset) for the *S. aureus* lipid film on biopolymers-containing subphases.

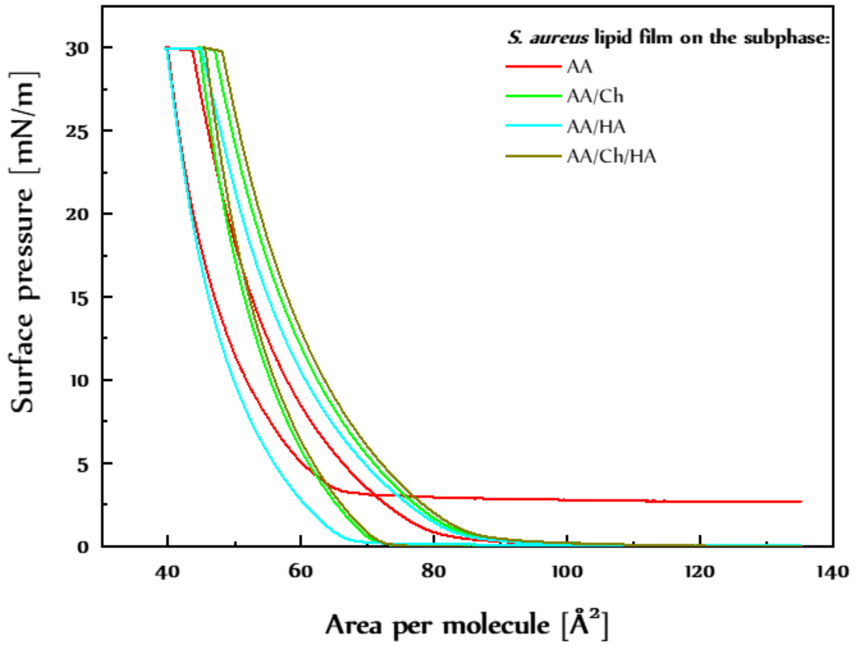

Figure S3. Hysteresis loops obtained by compression and decompression of the *S. aureus* lipid film on biopolymers-containing subphases.

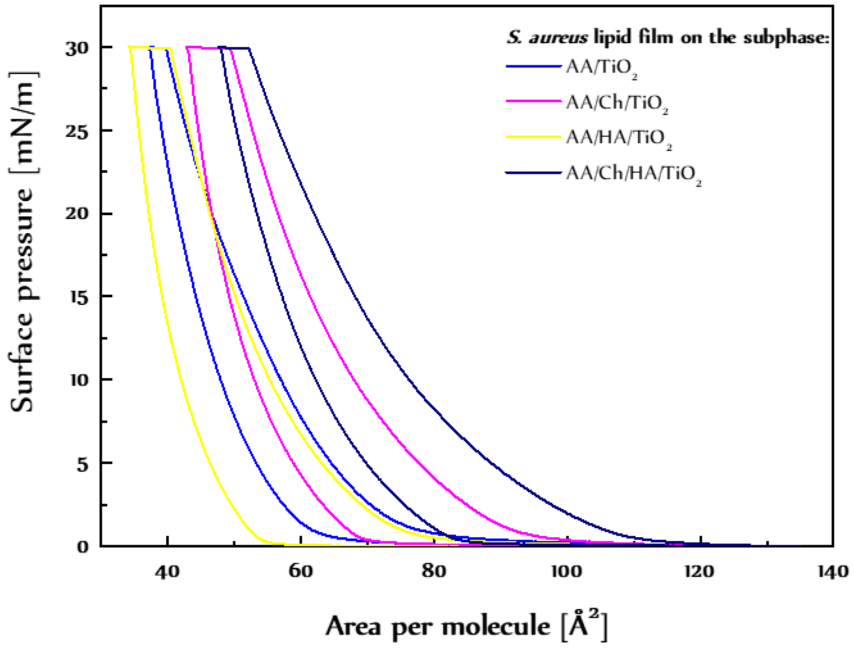

Figure S4. Hysteresis loops obtained by compression and decompression of the *S. aureus* lipid film on TiO_2-containing subphases.

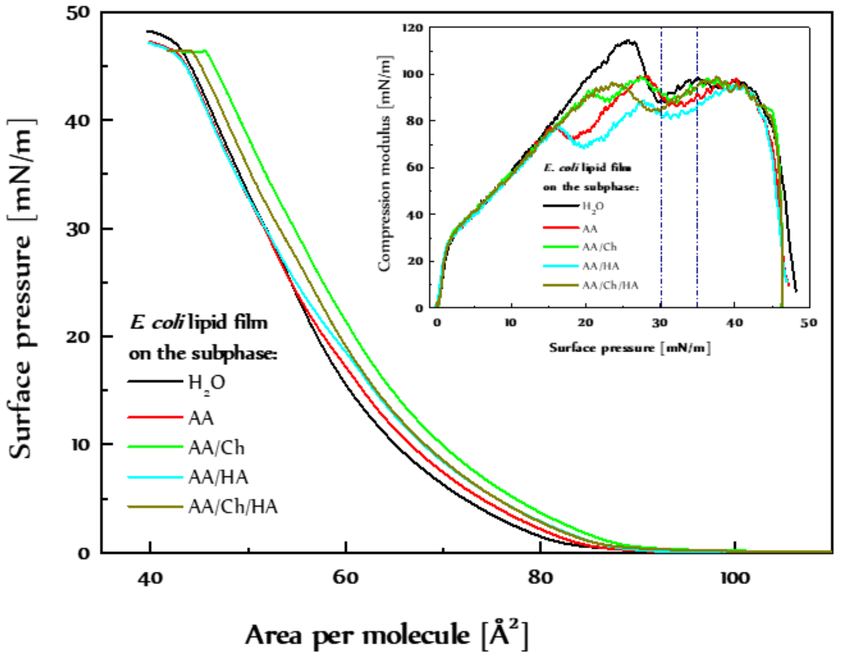

Figure S5. π-A isotherms obtained by means of the Langmuir technique and determined based on them Cs^{-1} =f(π) relationships (inset) for the *E. coli* lipid film on biopolymers-containing subphases.

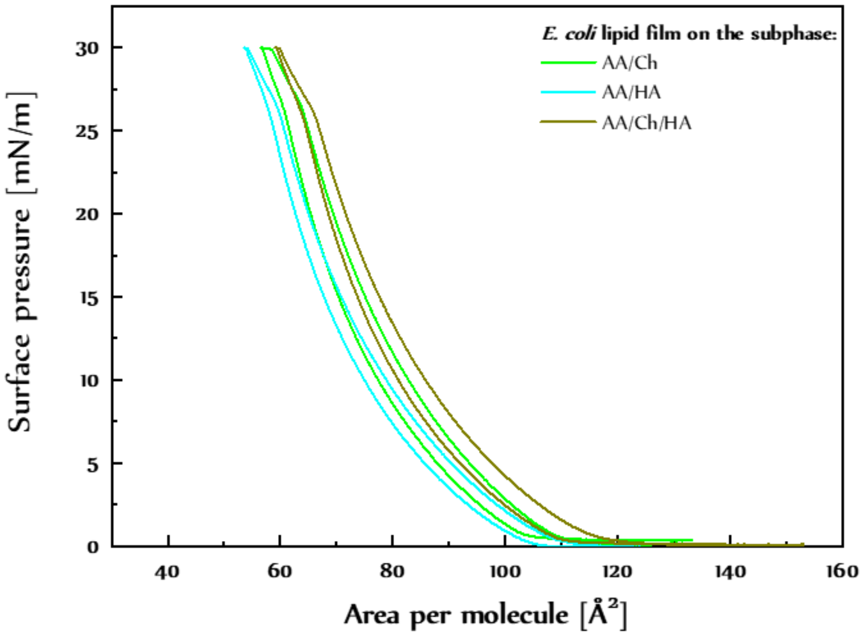

Figure S6. Hysteresis loops obtained by compression and decompression of the *E. coli* lipid film on biopolymers-containing subphases.

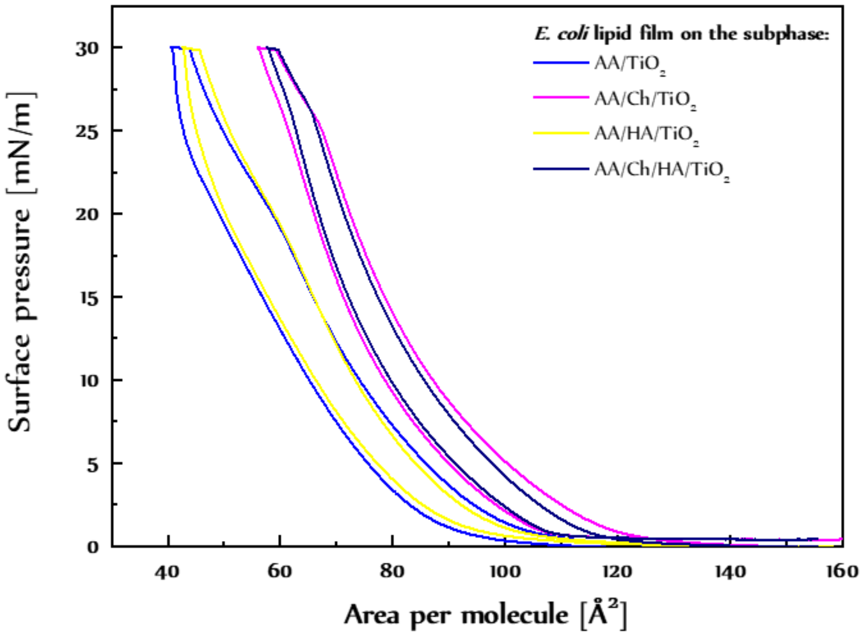

Figure S7. Hysteresis loops obtained by compression and decompression of the *E. coli* lipid film on TiO_2-containing subphases.
